# Woman and girl-centred care for those affected by female genital mutilation: a scoping review of provider tools and guidelines

**DOI:** 10.1186/s12978-022-01356-3

**Published:** 2022-02-22

**Authors:** Angela Dawson, Anisa Assifi, Sabera Turkmani

**Affiliations:** 1 Australian Centre for Public and Population Health Research, Faculty of Health University of Technology, Sydney, Australia; 2 Department of General Practice, Monash University, Melbourne, Australia

**Keywords:** Female genital mutilation, Women-centred, Girl-centred, Patient-centred, Clinical guidelines, Safeguarding

## Abstract

**Background:**

A woman and girl centred, rights-based approach to health care is critical to achieving sexual and reproductive health. However, women with female genital mutilation in high-income countries have been found to receive sub-optimal care. This study examined documents guiding clinicians in health and community service settings in English-speaking high-income countries to identify approaches to ensure quality women and girl-centred care for those with or at risk of female genital mutilation.

**Method:**

We undertook a scoping review using the integrative model of patient-centredness to identify principles, enablers, and activities to facilitate woman and girl-centred care interactions. We developed an inclusion criterion to identify documents such as guidance statements and tools and technical guidelines, procedural documents and clinical practice guidelines. We searched the databases and websites of health professional associations, ministries of health, hospitals, national, state and local government and non-government organisations working in female genital mutilation in the United Kingdom, Ireland, Canada, The United States, New Zealand, and Australia. The Appraisal of Guidelines for Research and Evaluation tool was used to appraise screened documents.

**Findings:**

One-hundred and twenty-four documents were included in this scoping review; 88 were developed in the United Kingdom, 20 in Australia, nine in the United States, three in Canada, two in New Zealand and two in Ireland. The focus of documents from the United Kingdom on multi-professional safeguarding (62), while those retrieved from Australia, Canada, Ireland, New Zealand and the US focused on clinical practice. Twelve percent of the included documents contained references to all principles of patient-centred care, and only one document spoke to all principles, enablers and activities.

**Conclusion:**

This study demonstrates the need to improve the female genital mutilation-related guidance provided to professionals to care for and protect women and girls. Professionals need to involve women and girls with or at risk of female genital mutilation in the co-design of guidelines and tools and evaluation of them and the co-production of health care.

**Supplementary Information:**

The online version contains supplementary material available at 10.1186/s12978-022-01356-3.

## Plain Language Summary

High-quality health care for women and girls should be provided by health workers who are respectful and caring. Health workers should also work with others as a team and help women and girls make their own decisions about their health care. This approach is called patient-centred care. Female genital mutilation (FGM) is a cultural practice associated with poor health outcomes. Women who have experienced FGM have not always received quality health care. We studied the guidance and tools to help health workers provide care to women and girls with or at risk of FGM. This study aimed to understand how health workers are supported to provide woman and girl-centred health care in these documents. We searched for these documents on the websites of health professional associations, government health departments and organisations working in FGM in the United Kingdom, Ireland, Canada, The United States, New Zealand, and Australia. The documents were analysed using a tool that described all the important areas needed to provide patient-centred health care. Of the 124 documents, we found that only 12% contained information about all aspects of patient-centred care. Only one document had details about all the areas that are part of patient-centred care. This study shows that we need to improve the FGM-related guidance provided to health workers to care for and protect women and girls. There is a need for health workers to involve women and girls with or at risk of FGM in designing guidelines and tools and evaluating them to these documents best fit their needs.

**Supplementary Information:**

The online version contains supplementary material available at 10.1186/s12978-022-01356-3.

## Background

Providing high-quality care for vulnerable women and girls, including those with female genital mutilation (FGM) or at risk of FGM 1, is an objective of health systems [1]. A woman and girl centred, rights-based approach to health care is central to achieving sexual and reproductive health [2]. However, women with FGM in high-income countries (HIC) have been found to receive sub-optimal care [3], report poor experiences of care [4] and health professionals have noted challenges caring for women and girls with FGM [5], indicating that much is needed to improve interactions between these women and their providers.

FGM is a deeply rooted cultural practice involving removing or modifying parts of the vulva that includes the opening of the vagina (vestibule), the labia majora, the labia minora, and the clitoris. The practice has no health benefits and is associated with adverse outcomes, including obstructed birth and negative effects on a woman’s mental and sexual health [6]. Migration from countries in Africa, Asia and the Middle East where FGM is practised traditionally has meant that clinicians in HIC are increasingly caring for affected women, counselling for prevention and reporting girls at risk due to the illegal status of FGM [7].

Woman-centred health care embodies feminist principles of empowerment by focusing on the individual needs of each woman [8] and is central to the World Health Organization’s (WHO) clinical guidelines on FGM [6]. However, the term girl-centred care is rarely used [9], while adolescent-centred [10, 11] and adolescent-friendly [12] are applied in clinical and service settings. These terms embody the concept of autonomy, respecting the wishes and values of the health service user and involving people in their care decisions that are aligned with patient-centred care [13], people-centred health services [14] and consumer participation [15]. These principles move health care from a standardised or disease‐oriented model to a more holistic, tailored partnership approach requiring a shift in power during interactions between what is traditionally referred to as clinicians and patients.

The Institute of Medicine in the United States asserts that putting people at the centre of their health care leads to more satisfactory, safer, higher quality care and improved health outcomes [13]. Systematic reviews have shown promising effects of patient-centred care on chronic disease management [16] and increased patient self-esteem and independence [17]. However, the impact of patient-centred care interventions on patient satisfaction, health behaviour and health status are mixed requiring, further research [18] Patient-centred care is supported by national and state directives such as the Australian National Safety and Quality Framework [19], The British Columbia Patient-Centred Care Framework in Canada [20], the National Health Service in the United Kingdom [21] and the US Veteran Health Administration [22] and is linked to service performance and funding.

Several studies have attempted to define care that places women, patients, people and consumers at the centre. Authors have described a continuum from authoritative disease-focused care or personalised medicine, where the patient is provided with educational materials, to patient-centred care where people are engaged in conversations, to person-centred involving the co-design and co-production of services [23]. While a recent review describes similarities across these concepts it found that the goal of person-centred care is a meaningful life, while the goal of patient-centred care is a functional life [24]. The latter emphasises power differentials through the use of the term patient and has been largely applied to medical contexts. However, even within the concept of consumer participation researchers have outlined levels of participation that depend on the input consumers have into service decision making [25]. Consumer participation appears to be central to mental health and drug and alcohol services [26]. Core components of women-centred care identified in the literature have focused on midwifery [27] and explored care for depression and cardiac rehabilitation [28]. There currently is no comprehensive model of women or girl centred care in the literature that has been applied across health contexts.

The patient-centred care literature provides the most detailed analysis of dimensions in broad health contexts that could be generally applied to women’s health and in particular, the delivery of women-centred care to women and girls with FGM or at risk of this practice. Langberg and others have recently identified core dimensions: biopsychosocial, patient-as-person, sharing power and responsibility, therapeutic alliance and co-ordinated care [29]. In line with these are 15 dimensions of patient-centeredness (grouped according to: principles, enablers and activities) identified in a systematic review of the literature by Scholl et al. [30]. The majority of these dimensions were later validated by a Delphi study that included patients [31]. The dimensions are essential characteristics of the clinician, clinician-patient relationship, clinician-patient communication, patient as a unique person, biopsychosocial perspective, patient information, patient involvement in care, involvement of family and friends, patient empowerment, physical support, emotional support, access to care, integration of medical and non-medical care, coordination and continuity of care, teamwork and teambuilding. These dimensions encompass those described by Brady et al. that comprise women-centred midwifery care [32] and six domains suggested by a Delphi survey of women and clinicians [33].

The domains identified by Scholl et al. help understand the quality of care interactions between a woman or girl and her provider and can be applied to the study and improvement of tools and guidelines developed to assist health care providers in facilitating women-centred care especially shared decision making, that constitutes an essential dimension described by Scholl et al. [30]. Several systematic reviews have explored how tools such as structured interview guides and infographics developed to increase women’s involvement in reproductive health decision making at the point of care have affected women’s knowledge, treatment choice and results, and women’s satisfaction. These include decision aides for heavy menstrual bleeding [34], abortion [35], contraception, vaginal birth after caesarean delivery, and pelvic organ prolapse [36]. While no studies have examined the use of such tools in shared decision-making in FGM contexts, several studies have examined how such tools have supported parent decision-making concerning male circumcision. In these contexts, studies have found that education materials have positively influenced communication and decision-making processes at the point of care [37, 38]. However, studies have noted that these processes did not affect preferences for circumcision of newborn male babies [39, 40].

Many tools and guidelines have been developed in HICs to support clinicians to better interact with women and girls with or at risk of FGM. These may include materials to prevent FGM, safeguarding guidance and checklists and tools to counsel pregnant women or presenting with gynaecological, mental health, or sexual health issues. However, there has been no examination of these to determine if they align with patient-centeredness dimensions. Previous systematic reviews have identified no papers providing insight into tools to be employed in high prevalence countries [41], including during counselling for deinfibulation [42]. However, numerous studies have identified poor clinician-patient communication and a lack of women’s involvement in maternity decision-making as central elements of women-centred care [4, 43, 44].

In response, we sought to investigate whether the tools and guidelines developed to ensure quality woman-centred care and support health providers to deliver this care align with patient-centred principles and provide opportunities to enable women-centred care. We undertook a review of statements, documents and tools to guide clinical practice to facilitate woman-centred care for women with FGM at the point of care in HIC. We sought to identify elements designed to facilitate woman-centred care in the guidance and tools provided to clinicians to interact with women who have FGM. According to evidence-based models, these insights can help identify approaches to ensure quality women and girl-centred care for those with or at risk of FGM.

## Methods

### Approach

We undertook a scoping review to map the key concepts underpinning the tools and guidelines developed to assist health professionals in caring for women and girls with FGM. In accordance with expert guidance on the use of scoping reviews, we aimed to provide a broad overview of the evidence of women and girl centred care in tools and guidelines that have been developed for use at the point of care and synthesise this knowledge to identify gaps, make recommendations for guideline improvements and future research [45, 46]. We applied this method in line with other scoping reviews examining clinical guidelines and best practice recommendations [47, 48].

We applied content analysis to produce rich descriptions of these policies, guidelines, procedures, and clinical practice guidelines (CPGs) to determine the extent to which they addressed women-centred care. We approached this study using the five-stage process for conducting a scoping review described by Levac et al. [49]. This included: developing the research question, identifying relevant studies, clarifying the study selection criteria, charting the data and finally reporting the results. This scoping review is registered as a project on the Open Science Framework http://dx.doi.org/10.17605/osf.io/agykd.

The key research question was: What guidance is provided to clinicians in English-speaking high-income countries to deliver patient-centred care to women and girls affected by or at risk of FGM? The analysis aimed to examine stated and unstated, tacit and implicit meanings and structures embedded within the documents. In the absence of a women and girl-centred model of care, we applied the integrative model of patient-centredness developed by Scholl et al. [30] as an apriori framework for the content analysis. We aimed to identify what activities were described in the documents to foster women-centred behaviour during encounters with clinicians. We also aimed to ascertain mechanisms that promoted women-centred principles in health service delivery, policy, regulation, and health and social care accreditation. Hence, we use the term women and girl centred care in this study to emphasise a holistic, tailored partnership approach requiring a shift in power during a range of health care interactions and to increase the visibility of women and girls.

### Search strategy

**Fig. 1:**
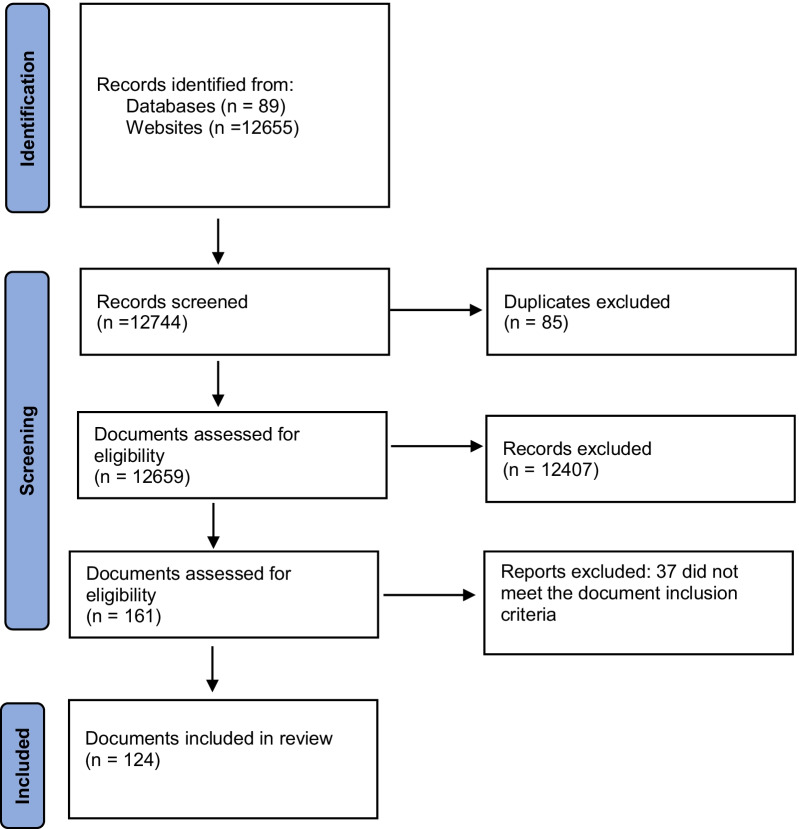
PRISMA flow diagram for new systematic reviews which included searches of databases and Websites

We searched the websites of health professional associations, ministries of health, tertiary hospitals, national, state and local government and non-government organisations working in the area of FGM in the United Kingdom, Ireland, Canada, The United States, New Zealand, and Australia. We searched datasets held by the European Union Open Data Portal, The Population Council’s Evidence to End FGM/C Programme database. We used the standard key words “female genital mutilation”, or “female genital cutting” or “FGM”. Electronic copies of all documents were downloaded and hard copies were obtained where this was not possible. Figure 1 outlines the search processes as per the PRISMA statement [50] and see Additional file 1 for details of websites and databases and numbers of documents returned, screened and included. We applied the “Identification of studies from other sources” approach [51] as this scoping review did not seek peer-reviewed research studies from traditional academic bibliographic databases, but rather websites and unique collections of documents from specific organisations. We used the PRISMA Extension for Scoping Reviews (PRISMA-ScR): Checklist to guide the reporting of this study [52].

### Eligibility criteria, study selection and quality assessment

The inclusion criteria used were the following: (i) documents included were guidance statements and tools (algorithms, flip charts, etc.), and technical guidelines, procedural documents and clinical practice guidelines (CPGs); (ii) documents had been published in the last 20 years (January 2001–December 2021); (iii) countries of origin were Anglosphere HIC settings as defined World Bank [53] and (iv) were in English, see Additional file 2. We defined health care broadly and included materials in English pertinent to medical, nursing, and allied health staff, as well as managers in high-income English-speaking countries who are recipients of migrants and refugees from countries where FGM is practised traditionally. We sought FGM-related documentation that focused on different levels of prevention, namely: primary, secondary and tertiary [54]. A ten-year time frame (since 2001) was selected to ensure the material was contemporary and clinically relevant. If documents had been updated, the latest version was chosen for inclusion.

A guideline was defined as a set of statements based on available information and best practice that provides health professionals (nurses, midwives, doctors, social workers and other allied health professionals) with appropriate options to manage specific issues, situations or circumstances. These guidelines can be clinical or corporate in their focus. We understood policy as a set of statements or intentions that indicate the position of a health services or organization related to FGM. A policy should guide conduct and decision-making and must be adhered to by employees. A policy is developed in response to a Board policy direction, a significant risk, a requirement of Government, a legislative requirement, or a significant community or local issue. Policy can be included in strategic documents, statements of priority and codes of conduct. Organisational policies must be endorsed by the Executive Committee and/or the Board of Directors of an organization. We sought to include procedural documents that we defined as containing instructions that specify ‘how’ to undertake a task. Procedures include instructions or steps to be followed to perform a task (e.g., de-infibulation, risk assessment) and are more prescriptive than a guideline. Finally, we searched for CPGs or documents outlining a set of statements that described best clinical practice based on a thorough evaluation of the evidence. Training materials were excluded as this study's focus was on examining available materials to support the delivery of care during a consultation. Training materials are developed to support the capacity building of health professionals to prepare them for health care interactions and are not usually designed for direct application at the point of care.

Two authors (AD and AA) screened the titles independently and excluded records that were not relevant. Disagreements were resolved with discussion. The same process was applied to the appraisal of the documents using the applied and validated assessment tool, the Appraisal of Guidelines for Research and Evaluation (AGREE) II, [55]. This comprises 23 items grouped into six domains and two overall assessment items. AD, AA and ST independently assessed 20 documents using the tool to establish agreement regarding the appraisal and rating of each item per domain. The three researchers then independently appraised every document. Domain scores were calculated by adding the three scores for the individual items in each domain per document and then scaling the total as a percentage of the maximum possible score for that domain as per the AGREE II guidelines. An overall quality rating was given to each guideline, considering the criteria considered in the assessment process and a recommendation made regarding the use of the guideline (see Additional file 3).

### Data extraction and analysis

The characteristics of documents were first plotted according to documentation type, context and intended use to determine patterns such as changes over time areas of focus and authors. According to the framework proposed by Scholl et al. [29], variables of interest were mapped to a table. This determined the initial coding scheme to examine relationships between codes. Text from the documents was then extracted and coded to an excel spreadsheet. We applied a directed content analysis [56] to validate and extend the framework proposed by Scholl et al. [30] to focus on women and girl-centred care interactions with health professionals that may include clinical and promotive interventions in community primary or tertiary care hospital contexts. Coding of relevant extracted data from the documents was independently undertaken by AA and AD; consensus was reached through discussion where there was disagreement. We then applied the integrative model of patient-centeredness to six documents that demonstrated the four fundamental propositions. A thematic analysis was then performed within the extracted data and consensus was reached among the authors.

### Findings

One-hundred and twenty-four documents were included in this scoping review (see Additional file 4), 88 were developed in the United Kingdom (UK), 20 in Australia, nine in the United States of America (US), three in Canada, two in New Zealand and two in Ireland. The year of the greatest number of publications was 2016 with 31 documents, followed by 2017 (26 documents), 2018 (15 documents), 2019 (10), 2015 (8), 2011 (8), 2013 (5). Fifty-three of the documents from the UK focused explicitly on safeguarding, with a further nine including safeguarding in guidance concerning the delivery of clinical care and recording FGM in medical records. The focus of documents from Australia, Canada, Ireland, New Zealand and the US was clinical practice, with two Australian documents including guidance related to safeguarding. The safeguarding documents from the UK emphasise multi agency and professional approaches across the health, education, community and justice sectors. The documents are aimed at a range of health professions with specific clinical and safeguarding guidelines and professional statement documents targeting nurses [57], midwives [58], obstetricians and gynaecologists [59–61], obstetricians and midwives [62–64], nurses and midwives [65–68], general practitioners [69, 70], family medicine specialists [71], physicians [70, 72], paediatricians [73, 74], paediatricians, obstetricians, gynaecologists and family medicine specialists [75], emergency medicine specialists [76], pharmacists [77], psychologists and counsellors [78], social workers [79–81].

We identified evidence of the 15 dimensions of patient-centredness (PC) described by Scholl et al. in the guidelines included in this review across three domains. These domains are namely principles (fundamental propositions, which lay the foundations for patient-centred care, enablers (elements, which foster patient-centred care), and activities (specific patient-centred behaviour) see Tables 1 and 2.

**Table 1:** Guidelines and tools that demonstrate propositions that are consistent with all four principles of patient-centredness

	Principles				Enablers					Activities					
Reference & notes	Essential characteristics of the clinician	Clinician-patient relation-ship	Patient as a unique person	Bio-psycho-social perspective	Clinician-patient communication	Integration of medical & non-medical care	Team work	Access to care	Coordination & continuity of care	Patient inform-ation	Patient involvement in care	Involve-ment of family & friends	Patient empowerment	Physical Support	Emotional support
Australia															
[82] Safeguarding & care guide	✓	✓	✓	✓	✓				✓	✓		✓			
[151] Service co-ordination guide that includes the flow chart outlining care	✓	✓	✓	✓	✓			✓	✓	✓					
[84] Care of women with FGM	✓	✓	✓	✓	✓			✓	✓	✓		✓			
[85] Includes Practice Guidelines	✓	✓	✓	✓	✓		✓	✓	✓	✓	✓	✓	✓	✓	✓
Canada															
[75] Clinical practice guideline developed by multiple professional committees	✓	✓	✓	✓	✓		✓	✓	✓	✓	✓	✓		✓	✓
Ireland															
[152] Handbook for health professionals	✓	✓	✓	✓	✓				✓	✓		✓		✓	✓
UK															
[86] Multi-agency guidance talking about FGM & appropriate professional response	✓	✓	✓	✓			✓								
[153] Multi-agency statutory guidance—safeguarding	✓	✓	✓	✓	✓		✓	✓	✓	✓	✓	✓		✓	✓
[88] Health visitors & school nurses mandatory reporting	✓	✓	✓	✓	✓		✓	✓	✓	✓	✓	✓	✓		✓
[89] Multi-agency pathways, screening, risk assessment checklist tools & referral flowcharts for women & girls	✓	✓	✓	✓	✓		✓	✓	✓	✓		✓		✓	✓
[154] Clinical standards for services includes referral & safeguarding, data collection & documentation	✓	✓	✓	✓	✓			✓	✓	✓				✓	✓
[68] Resource for nursing and midwifery practice	✓	✓	✓	✓	✓		✓	✓	✓	✓	✓	✓			
[155] Toolkit How to do direct work on FGM with children, young people, parents and carers for Social Workers	✓	✓	✓	✓	✓		✓			✓	✓	✓	✓		
[156] Working Therapeutically with Survivors counsellors, psychologists	✓	✓	✓	✓	✓	✓	✓	✓	✓	✓	✓	✓	✓	✓	✓
US															
[92] Clinical report guidance for the clinician in paediatric care	✓	✓	✓	✓	✓		✓		✓	✓	✓	✓		✓	✓

**Table 2:** Frequency of dimensions of patient centredness across the included documents

Principles	Number of documents
Essential characteristics of the clinician	70
Clinician-patient relationship	31
Patient as a unique person	38
Bio-psycho-social perspective	60
Enablers	
Clinician-patient communication	97
Integration of medical & non-medical care	1
Team work	72
Access to care	31
Coordination & continuity of care	77
Activities	
Patient information	71
Patient involvement in care	27
Involvement of family & friends	44
Patient empowerment	9
Physical Support	19
Emotional support	40

Fifteen guidelines and tools contained information outlining fundamental propositions that are consistent with all four principles of PC [68, 74, 75, 78, 81–93] (Table 1). Of these documents, four were CPGs, four were guidelines or manuals for service providers, three were guidelines for service co-coordinators, and five were concerned with safeguarding and mandatory reporting at local and national government levels. Only one of these fifteen documents, a guide designed for counsellors and psychologists to work therapeutically with survivors of FGM by Coho et al. contained examples of all the principles, enablers and activities of PC [78].

Across all 124 documents, the 15 dimensions of PC the enabler, clinician-patient communication featured as the most common advice to health professionals (78% or 97 documents see Table 2). Of the other enablers, teamwork was included in 72 documents and coordination and continuity of care in 77 documents, just over half of the documents. The essential characteristics of the clinician featured in 70 documents while the activity, the provision of patient information, was identified in 71 documents. The remaining dimensions were identified in less than 50% of the included documents.

### Principles

The 70 documents that provided directions to professionals concerning the essential characteristic required for working with women and girls with or at risk of FGM featured the need for sensitive and non-judgemental interaction that avoids stigmatising the woman or girl. Professional behaviour was emphasised in relation to safeguarding [94]. In addition, documents suggested health professionals be educated, confident and prepared so “that they do not exhibit signs of shock, confusion, horror or revulsion on seeing the genitalia” [95].

The 31 documents that discussed the clinician-patient relationship emphasised the need to develop “trusting relationships” [75, 85, 88, 92], and “rapport” [87, 93, 96] to “create [an] opportunity for the individual to disclose.” [97], “Make the woman/girl feel comfortable” [82] and to work “in collaborative partnerships” [83, 98] and enable a “a plan of care [to] be made in collaboration with the woman” [63]. Documents identified the need to “establish trust-based relationships that foster respectful, transparent, evidence-based care” [65] and that “Clinicians are also urged to be clear about your role, scope, authority and responsibility” [99].

The girl or woman was acknowledged as a unique person in 38 documents by ensuring that “the voice of the child is heard when discussing issues surrounding FGM “[100], a “victim-centred approach” [87] or “woman-centred care” [75] where “each case is considered individually” [92, 101], “as unique” [102] or “case by case” [89] to meet the needs of the woman or girl [61, 75, 86, 103, 104]. Documents identified a rights-based approach including the need for health professionals to “respect individual needs” [105, 106], “wishes” [107, 108], “privacy” [109, 110], “dignity” [93] and “preferences” [65, 88, 90] of girls and women. Documents call for professionals not to “make assumptions about a woman because of her religion or ethnicity” [82] and “adapt approaches accordingly” [80], taking into account “socio-cultural factors from both the client’s culture of origin and her place in the culture in which she currently resides” as well as how “Each client will have experienced this [FGM] trauma and its consequences in her own personal, unique way.” [78]. This also points to 60 documents that embraced a bio-psychosocial perspective that acknowledges physical, emotional and mental health and the need for “ holistic care” that involves “discussions about healthy choices” [75] and responds to women and girls culture and language, “health literacy” [84, 92] and the “intersection of ethnicity, migration, sex, and gender” [92]. Some documents specifically mentioned the need for care to be “culturally competent” [74, 75, 104] and recognise the trauma of FGM and refugee experience [83, 111].

### Enablers

As noted, the enabler “Clinician-patient communication” was most frequently identified dimension across all documents. Common to nearly all the 97 documents were the provision of advice about the need to use appropriate terminology for FGM (cutting, circumcision or words from local languages), the use of professional female interpreters, simple questions posed one at a time in a sensitive way and clear explanations including regarding the types of FGM, deinfibulation, the law and, the importance of listening and allowing time for the girl and woman to talk and checking for understanding. “Open and honest” [100], culturally sensitive [90], supportive and compassionate [112], uninterrupted [87] communication is encouraged along with a reminder about the complex nature of the topic, that this may be the first time a woman has discussed FGM with another person [113] and that in safeguarding contexts the conversation must be conducted alone and in private to assist the individual to disclose [114]. Providers are instructed to prepare conversations in line with the possibility that “a girl may be embarrassed” [88], or that a woman “may be distressed at the suggestion that she would do the same to her daughter” [115]. Many documents provide examples of questions to ask [96, 116], with some aligned with various templates to assess risk for safeguarding and obstetric risk [117–119] and encourage asking supportive open questions [120] that incorporate the use of the 4Cs in conversations (confidence, caring, client-centred and collaboration) recommended by Raymond [121]. Professionals are reminded to “stick to facts e.g., the legal position and health implication” [122] and be mindful of their body language including maintaining eye contact [123, 124] and communication barriers including “literacy, learning disabilities, cultural considerations and English as a second language” [80].

Only one document contained the dimension; the integration of medical and non-medical care and the sentence: “Respectfully enquire about traditional healing practices, including any potential secondary effects” was coded to this dimension [78]. Guidance on teamwork was mainly provided in the form of multi-agency safeguarding and risk assessment protocols in the British documents. Other documents suggest working with professionals who have expertise interacting with women and communities in relation to FGM, conducting regular multi-disciplinary meetings, and ensuring records or documentation to facilitate collaboration [123]. This assists women in accessing services by supporting her to navigate the health system and facilitate co-ordinated efforts to ensure referral and continuity of care to meet her needs [75, 92].

### Activities

Examples of activities to achieve PC across six dimensions were identified in most documents. The provision of various types of patient culturally sensitive information was most common including proposing a birth and deinfibulation plan [93], information about the law and the adverse effects of FGM, available health and social services, education on healthy body image, sexual and reproductive health and managing long-term complications [71]. Many documents suggested resources such as pamphlets or leaflets in relevant languages that included diagrams to explain the types of FGM [75, 91, 92, 125]. The involvement of women and girls in their care was suggested by gaining consent in safeguarding interactions [126] and the development of a plan for birth in “partnership with the woman” [98] or “shared”, “informed” and “mutual” decision-making about deinfibulation and other care [68, 75, 88, 127, 128] along with references resources to support clinicians with this process [87]. The involvement of husbands and partners in clinical care decision making [85] was highlighted and the need to offer them counselling [115], observe for possible coercion [129] and provide letters to parents concerning the illegal nature of FGM, or running coffee morning or workshops with them on this topic before holiday periods [101]. One document identified involving women’s health advocates in consultations who improve service uptake and can also provide links to the community for the distribution of education [90].

Activities to describe empowering women and girls were noted in only nine documents. Suggestions included arranging meetings with the parents and teenagers with FGM to enhance health literacy and self-efficacy to seek care when needed [92] or ensure midwives lead conversations to enable women to build an understanding of how to “take care of themselves” [85]. Other guides suggested identifying “community assets” to develop education to prevent FGM [88] or using a genogram activity to map family relationships to identify where “social care concerns lie and who may be able to assist in the protection of the girl within a family” [80]. Fewer documents (19) guided the promotion of physical support for those with FGM (primarily pain relief options) as opposed to emotional support (40 documents) via the provision of psychological counselling.

#### Appraisal of the documents

Additional file 3 summarizes the AGREE II appraisal of included guidelines. Forty documents (32%) were recommended with modification and 84 (68%) were not recommended. Scaled domain percentage scores varied widely across guidelines. Scope and purpose (100% to 5.6%), stakeholder involvement (100% to 0%), rigor of development (76.4% to 0%), clarity of presentation (100% to 16.7%), applicability (75% to 0%) and editorial independence (66.7% to 0%).

We were particularly interested in the second domain “Stakeholder involvement” and sought examples of how women’s experiences and voices had been integrated. Notable examples included one Australian guideline that quoted excerpts from a woman’s story highlighting how her wishes were met and how their preferences were accommodated [85]. This document recognised the input of a female interpreter and consumer. Another Australian document acknowledged that information contained in the guideline was also sourced from focus groups conducted with young African women [84]. While other documents did not state that they involve women or girls, they referred to consultation with NGOs that actively involved survivors [78, 92].

## Discussion

For the first time, this scoping review has collated all guidance for health professionals in HIC English-speaking countries to care for and protect women and girls with or at risk of FGM. We applied the integrative model of PC to identify principles, enablers and activities to facilitate woman and girl-centred care interactions. While we only identified one document that provided all examples of the 15 dimensions, we could clarify examples from all dimensions that can be used to update or develop new guidance for clinical care and safeguarding. Most documents focused on reminding clinicians of the importance of being a respectful professional, using appropriate communication skills, working in teams, and providing relevant information. Despite acknowledging a bio-psychosocial perspective in many documents, only one reference was made to complementary or traditional medicine, and the empowerment and active involvement of women and girls in decisions concerning their care featured in less than 20 percent of documents. This indicates that there are many opportunities to enhance guidance to improve health professionals’ ability to recognise the unique experience of women and girls and develop rapport to share power and responsibility and find common ground for understanding and agreement. The gaps we found in patient centred care (PCC) for women concur with another study [28]. However, this research did not identify a guideline that covered all patient-centred domains for women based on the domain described by McCormack et al. [130].

### Guidelines that support the involvement of women and girls in their care

In our study, patient involvement in care was often restricted to gaining the consent of women or girls in the documents, particularly those concerned with safeguarding rather than shared decision making. This is closely related to patient empowerment or building self-efficacy to enable a woman or girl to self-manage aspects of their health such as, engaging in a mutual support group or an education programme. However, only nine documents made specific references to this element of PC. The WHO Clinical Handbook emphasises the provision of choice and autonomy to allow women and girls to make an informed decision and lays out an approach to developing a care plan with them that incorporates individual preferences [6].

Future guideline developers may wish to examine the WHO Handbook and look to decision-making guidance and tools that foster an approach to collaborative deliberation to promote dialogue and increased joint discussion [131]. Such tools have demonstrated a positive effect on the health outcomes of disadvantaged patients [132]. However, these tools and guidance must be informed by understanding the women’s socio-cultural context and her goals, values, and preferences for health and demand women’s involvement in their development.

### The co-design of women and girl-centred guidelines, policies and tools

Our appraisal found little evidence of the direct involvement of women and girls, who are migrants or refugees from countries where FGM is traditionally practised, in developing or co-designing these tools. There has been a lack of research into the involvement of refugee and migrant women and girls in identifying important elements of PC. A scoping review [133] examining research on the enablers and barriers to PC for migrants and refugees identified that women (some from FGM prevalent nations) appreciated clinicians who were non-judgemental, clinically competent, provided time to ask questions and appropriate information to enable shared decision making. In addition, women valued female providers from the same culture or religion. These enablers can be mapped to the principle of “essential characteristics of the clinician”, the enabler “clinician-patient communication” and the activity dimensions of “Patient information” and “patient involvement in care”. These dimensions have also been identified in qualitative research with women, ten of these studies originated from countries where FGM is practised [134].

While little is known about how women and girl migrants and refugees understand PC and what they value, few studies have examined how these dimensions can be harnessed in co-design efforts with this population. Co-design can produce guidelines and tool that can facilitate effective communication between a woman or girl and her provider to develop a shared understanding of an issue and generate a mutually acceptable evaluation and management plan if required. Research with 50 participants (managers, clinicians and patients) has investigated ways to incorporate patient preferences in guidelines [135]. However, women and girl migrants and refugees were not a feature of this work.

Our study could not identify how research on delivering quality care to women with FGM has informed the included documents. For example, a study by Jacoby [136] found that the timing of the use of a co-designed comic book style health education tool to improve communication with Somali women and their understanding of perinatal health, including emergency caesareans and postpartum depression early in the antepartum period was more effective than late counselling. This tool was validated as useful by these women who had experienced FGM, but the early timing was preferred as it gave them sufficient time for thinking and discussing health concerns with their husbands.

### The effectiveness of guidelines and tools to enhance women and girl-centred care

No studies examine the effectiveness of guidelines and tools to promote women and girl-centred reproductive care in practice. Studies have instead focused on the impact of decision aids on a woman’s informed decision-making [36]. The success of guidelines and tools will depend on the involvement of women and girls in their development and the consideration of all dimensions and how autonomous women and girls are to engage in a patient-centred encounter. Some research has examined how patient empowerment can be effectively measured to enable health professionals to provide capacity-building support in reproductive health interactions [137]. PCC may need to be adjusted according to the level of a woman’s autonomy and empowerment. Women with FGM are often in a very disempowered position due to low English language and health literacy skills and experience fear and anxiety related to the stigma of FGM, racism and discrimination [4].

Assessing the outcomes of the use of PC guidelines for women and girls with or at risk of FGM may require the development of measures including access to care and patient-reported outcomes. Ideally, women and girls should be involved in evaluating such guidelines and the development of these measures. Generic patient-centred quality indicators have been identified from a systematic review [138] across the organisational level of the health care system, during the process of the patient-provider interaction and at the outcome of the consultation. This model includes the importance of supporting the workforce to deliver PCC and providing an environment conducive to this.

### Preparing and supporting clinicians to deliver women and girl-centred care

Santana et al. [139] identified the importance of building the capacity needs of health professionals to deliver PCC through in-service education, ongoing professional development, supervision and performance management. Person-centred principles should also be clearly outlined in position descriptions and providers should be able to articulate PC practices applicable to their role(s) and demonstrate their implementation.

There are many training programmes to support the in-service needs of clinicians to care for women and girls with or at risk of FGM that focus on building cultural competency and communication skills for FGM consultations [139, 140] and supporting professionals to prepare for a safeguarding interaction [141, 142]. Guidance has also been provided for training medical, nursing and midwifery students [143, 144]. Evaluations of FGM training programmes have not clarified how this has translated to PCC in practice [145–147]. However, studies in other areas have found that training and guidance to support PCC has had some success in increasing medical residents’ empathy scores [148]. Still, a recent analysis of the medical curriculum found that PCC was rarely noted in the documents [149].

### Implementing women and girl-centred care

Ensuring the comprehensive implementation of FGM women and girl-centred guidelines and care will require the collective efforts of health services and related organisations across the whole health system. Our study found that there is a strong focus in the UK on multi-agency guidelines and documents to support practice at various administrative (councils, local governments, boroughs and combined authorities), jurisdictional (England, Scotland and Wales), and national levels (UK). Australia similarly has state and territory guidelines but no national ones. We located guidelines for commissioners of health services in the UK, CPGs and policies issued by hospitals, professional associations and national centres of excellence. Despite this coverage, our appraisal of the included documents identified few that provided commentary on how they could be implemented, including the barriers, resource implications, and monitoring requirements. This is likely to constrain the implementation of PCC in organisational contexts. A survey of various health and social care organisations in Germany identified a wide range of determinants across multiple dimensions affecting PCC [150] that may also apply to the provision of care for women with or at risk of FGM. Hower et al. found that the active involvement of managers and decision-makers was critical to ensure that the priorities and values of organisations were in the appropriate position to address financial, human and material resources constraints required to deliver PCC.

## Limitations

This study is limited by its largely descriptive nature and as such is only able to provide an overview of available guidance statements and tools and technical guidelines, procedural documents and CPGs. The documents reviewed in this study were limited to those publicly available online. Documents on member only sites and intranets could not be included. While the authors endeavoured to search all known relevant databases and websites in the six countries, selection bias is a possibility if data was missed, affecting the descriptive account of available information.

## Conclusion

In our study, 15 of the 124 included documents included references to all principles of PC, and only one document spoke to all enablers and activities. These findings point to the need to improve the FGM-related guidance provided to health professionals to care for and protect women and girls. This research points to the need for health professionals to involve women and girls with or at risk of FGM in co-designing guidelines and tools and evaluating them and the co-production of health care. Delivering women and girl- centred health care to this unique population will require re-orientating the model of care so that women and girls can contribute to the provision of health services as partners of professional providers. However, ensuring that all consultations are women and girl-centred requires building the workforce's capacity and supportive leadership and governance to provide financial and policy resources.

## Supplementary Information

**Additional file 1: **Search Strategy.

**Additional file 2: **Inclusion and exclusion criteria.

**Additional file 3: **Quality of included guidelines appraised with AGREE II.

**Additional file 4: **All documents included in the scoping review mapped according to the dimension of patient centred care.

## Data Availability

All data is available upon request.

## Abbreviations

FGM: Female Genital Mutilation
PC: Patient-centredness
PC: Patient centred care
UK: United Kingdom
UN: United Nations
US: United States of America
WHO: World Health Organization

## Footnotes

1 The term Female Genital Mutilation is used in this study as defined by the WHO to reinforce “the fact that the practice is a serious violation of girls’ and women’s rights. This term also establishes a clear distinction from male circumcision, and emphasises the gravity and harmfulness of the act.” [6]. FGM is a term used in professional settings, however we acknowledge that interactions with women, girls and their families must involve acceptable and non-judgemental language. Health professionals are encouraged to use terms such as female genital cutting (FGC) excision or genital cutting, as well as terms from the languages of practising communities such as “sunna”
