# Supplementary material for: Woman and girl-centred care for those affected by female genital mutilation: a scoping review of provider tools and guidelines
Source: Reprod Health. 2022 Feb 22;19:50. doi: 10.1186/s12978-022-01356-3 (PMC8862274; doi:10.1186/s12978-022-01356-3)
Supplement: Supplementary file 1 — Additional file 1: Search Strategy. [file 12978_2022_1356_MOESM1_ESM.docx]

**Additional file 1. Search Strategy**

| **Data Europa Database** | |  |
| --- | --- | --- |
| Search for datasets containing FGM 8 returned and 3 appropriate <https://data.europa.eu/euodp/data/dataset?q=FGM&ext_boolean=all&sort>= | | |
|  | Items returned | Items included |
| [**Good practices in combating Female Genital Mutilation**](https://data.europa.eu/euodp/data/dataset/good-practices-in-combating-fgm) | 3 | 0 |
| [**Methods and tools on combating Female Genital Mutilation**](https://data.europa.eu/euodp/data/dataset/methods-and-tools-on-combating-female-genital-mutilation) | 22 | 0 |
| [**Resources on Female Genital Mutilation**](https://data.europa.eu/euodp/data/dataset/resources-on-fgm) | 1 | 0 |
| **Evidence to End FGM Database** <http://www.evidencetoendfgmc.org/> | |  |
| <http://www.evidencetoendfgmc.org/index.php/searchprocess?gw_program=&gw_region=&gw_category=Medical+and+psychosocial+support&gw_audience=&search>= | | |
| **Search terms** | Items returned | Items included |
| Medical and psychosocial support | 5 | 0 |
| United Kingdom + intervention | 42 | 3 |
| Canada + intervention | 5 | 0 |
| New Zealand + intervention | 1 | 1 |
| Ireland + intervention | 5 | 0 |
| Scotland + intervention | 1 | 0 |
| USA + intervention | 4 | 0 |
| **Total** | **89** | **4** |
|  |  |  |
| **Websites** | | |
| **Australia: Websites and search terms** FGM/ female genital mutilation/ female genital cutting |  |  |
| [Australian Government Department of Health https://www.health.gov.au/](Australian Government Department of Health https://www.health.gov.au/ ) | 108 | 0 |
| NSW Health <https://www.health.nsw.gov.au/> | 196 | 2 |
| Queensland Health <https://www.health.qld.gov.au/> | 228 | 1 |
| Northern Territory Health <https://health.nt.gov.au/> | 5 | 1 |
| Tasmanian Department of Health <https://www.health.tas.gov.au/> | 55 | 1 |
| Western Australia Department of Health <https://ww2.health.wa.gov.au/> | 50 | 0 |
| Department of Health Victoria <https://www.health.vic.gov.au/> | 1 | 0 |
| South Australia Health <https://www.sahealth.sa.gov.au> | 20 | 1 |
| Australian Capital Territory Department of Health <https://health.act.gov.au/> | 10 | 1 |
| Multicultural Centre for Women’s Health <https://www.mcwh.com.au/> | 41 | 0 |
| National Education Toolkit for Female Genital Mutilation/Cutting Awareness <https://netfa.com.au/resources/category/professionals/> | 41 | 8 |
| Women’s Health West <https://whwest.org.au/single/manuals-guides-tools/> | 12 | 2 |
| Cohealth - Community Health Service  <https://www.cohealth.org.au/> | 2 | 1 |
| Family Planning NSW <https://www.fpnsw.org.au/> | 4 | 0 |
| SHINE SA <https://shinesa.org.au/> | 1 | 1 |
| SASHA: South Australian Sexual Health Awareness https://sasha.shinesa.org.au/ | 6 | 2 |
| Family Planning Victoria <https://www.fpv.org.au/> | 42 | 2 |
| True <https://www.true.org.au/> | 4 | 0 |
| King Edward Hospital <https://www.kemh.health.wa.gov.au> | 31 | 2 |
| The Royal Women’s Hospital <https://www.thewomens.org.au/> | 7 | 1 |
| Australian College of Nursing <https://www.acn.edu.au/> | 4 | 0 |
| Australian College of Midwives <https://www.midwives.org.au/> | 7 | 0 |
| Royal Australian College of General Practitioners (RACGP) <https://www.racgp.org.au/> | 183 | 1 |
| Royal Australian and New Zealand College of Obstetricians and Gynaecologists <https://ranzcog.edu.au/> | 4 | 1 |
| Royal Australasian College of Physicians <https://www.racp.edu.au/> | 18 | 1 |
| The Royal Australian and New Zealand College of Psychiatrists <https://www.ranzcp.org/home> | 3 | 0 |
| Australian Medical Association <https://www.ama.com.au/> | 46 | 1 |
| Australian Psychological Society <https://psychology.org.au/> | 57 | 0 |
| **Total** | **1186** | **35** |
| Duplicates removed |  | 9 |
| Removed at screening |  | 6 |
| **Included** |  | **20** |
|  |  |  |
| **Canada : Websites and search terms** FGM/ female genital mutilation/ female genital cutting |  |  |
| Caring for Kids New to Canada <https://www.kidsnewtocanada.ca/> | 72 | 3 |
| Health Canada <https://www.canada.ca/en/health-canada.html> | 423 | 4 |
| Ministry of Health Alberta <https://www.alberta.ca/health.aspx> | 4 | 0 |
| Ministry of Health British Columbia  <https://www2.gov.bc.ca/gov/content/governments/organizational-structure/ministries-organizations/ministries/health> | 7 | 0 |
| Manitoba Health and Seniors Care <https://www.gov.mb.ca/health/> | 0 | 0 |
| New Brunswick Health Department <https://www2.gnb.ca/content/gnb/en/departments/health.html> | 4 | 0 |
| Newfoundland and Labrador Department of Health and Community Services <https://www.gov.nl.ca/hcs/> | 0 | 0 |
| Nova Scotia Health Authority <http://www.nshealth.ca/> | 1 | 1 |
| Ministry of Health Ontario <https://www.ontario.ca/page/ministry-health> | 346 | 1 |
| Prince Edward Island Department of Health and Wellness <https://www.princeedwardisland.ca/en/topic/health-and-wellness> | 4 | 0 |
| Quebec Minister of Health and Social Services  <https://publications.msss.gouv.qc.ca/msss/en/recherche/?txt=female+genital+mutilation&rechercher=Start+search&msss_valpub>= | 0 | 0 |
| Ministry of Health Saskatchewan <https://www.saskatchewan.ca/government/government-structure/ministries/health> | 2 | 0 |
| Northwest Territories Department of Health and Social Services <https://www.nthssa.ca/en> | 0 | 0 |
| Nunavut Department of Health <https://www.gov.nu.ca/health> | 0 | 0 |
| Yukon Department of Health and Social Services <https://yukon.ca/en/department-health-social-services> | 556 | 0 |
| Canadian Association of Midwives <https://canadianmidwives.org/> | 0 | 0 |
| Canadian Nurses Association <https://www.cna-aiic.ca/en/home> | 6 | 0 |
| Society of Obstetricians and Gynaecologists of Canada <https://sogc.org/> | 0 | 0 |
| The College of Family Physicians of Canada <https://www.cfp.ca/> | 5 | 0 |
| Canadian Psychiatric Association <https://www.cpa-apc.org/> | 0 | 0 |
| Canadian Paediatric Society <https://cps.ca/> | 2 | 0 |
| The Royal College of Physicians and Surgeons of Canada <https://www.royalcollege.ca/rcsite/home-e> | 0 | 0 |
| Canadian Medical Association <https://www.cma.ca/> | 0 | 0 |
| Canadian Psychological Association <https://cpa.ca/> | 0 | 0 |
| **Total** | **1432** | **5** |
| Duplicates removed |  | 1 |
| Removed at screening |  | 1 |
| **Included** |  | **3** |
|  |  |  |
| **Ireland: Websites and search terms** FGM/ female genital mutilation/ female genital cutting |  |  |
| Department of Health Ireland <https://www.gov.ie/en/organisation/department-of-health/> | 4 | 0 |
| The Irish College of General Practitioners <https://www.icgp.ie/> | 10 | 1 |
| Institute of Obstetricians & Gynaecologists <https://www.rcpi.ie/faculties/obstetricians-and-gynaecologists/> | 1 | 1 |
| Irish Paediatric Association <http://www.irishpaediatricassociation.ie/> | 0 | 0 |
| Royal College of Surgeons in Ireland <https://www.rcsi.com/> | 10 | 0 |
| Irish Nurses and Midwives Organisation  <https://www.inmo.ie> | 1,180 | 1 |
| Irish Family Planning Association <https://www.ifpa.ie/> | 25 | 1 |
| AkiDwA <https://akidwa.ie/about-us/>  Publications <https://akidwa.ie/publications/?_sft_papers_cat=publications> | 11 | 1 |
| **Total** | **1241** | **5** |
| Duplicates removed |  | 3 |
| Removed at screening |  | 0 |
| **Included** |  | **2** |
|  |  |  |
| **New Zealand: Websites and search terms** FGM/ female genital mutilation/ female genital cutting |  |  |
| New Zealand Ministry of Health <https://www.health.govt.nz/our-work/populations/refugee-health/health-needs/physical-health> | 4 | 1 |
| **New Zealand FGM Education Programme** <https://fgm.co.nz/resources/health-professionals/> | 6 | 2 |
| Nursing Council of New Zealand <https://www.nursingcouncil.org.nz/> | 0 | 0 |
| New Zealand College of Midwives <https://www.midwife.org.nz/> | 1 | 0 |
| The Royal New Zealand College of General Practitioners <https://www.rnzcgp.org.nz/> | 0 | 0 |
| New Zealand Psychological Society  <https://www.psychology.org.nz/> | 0 | 0 |
| **Total** | **11** | **3** |
| Duplicates removed |  | 0 |
| Removed at screening |  | 1 |
| **Included** |  | **2** |
|  |  |  |
| **UK Websites and search terms** FGM/ female genital mutilation/ female genital cutting |  |  |
| Department of Health & Social Care <https://www.gov.uk>  <https://www.gov.uk/search/all?keywords=female%20genital%20mutilation&content_purpose_supergroup%5B%5D=guidance_and_regulation&order=relevance> | 603 | 46 |
| UK Government includes Homes Office, Public Health England, Department of Health <https://www.gov.uk/government/organisations/home-office> | 420 | 12 |
| Local Government Association <https://www.local.gov.uk/>  <https://www.local.gov.uk/topics/community-safety/female-genital-mutilation> | 7 | 2 |
| Google Search "Safeguarding Children Female genital mutilation" | 259 | 8 |
| National Society for the Prevention of Cruelty to Children <https://learning.nspcc.org.uk/safeguarding-child-protection> | 20 | 11 |
| NHS UK <https://www.nhs.uk/> | 3 | 1 |
| NHS England <https://www.england.nhs.uk/> | 385 | 21 |
| London boroughs <https://www.londoncouncils.gov.uk/> | 14 | 5 |
| NHS Wales <https://www.wales.nhs.uk/> | 194 | 1 |
| Local Government Wales <https://gov.wales> | 152 | 2 |
| NHS Scotland [www.publications.scot.nhs.uk](http://www.publications.scot.nhs.uk) | 4 | 2 |
| Public health Scotland <https://publichealthscotland.scot/> | 0 | 0 |
| Local government Scotland <https://www.gov.scot/policies/local-government/> | 42 | 2 |
| Health and Social Care Northern Ireland <http://online.hscni.net/> | 0 | 0 |
| Royal College of Obstetricians and Gynaecologists <https://www.rcog.org.uk/> | 69 | 2 |
| Royal College of Nursing <https://www.rcn.org.uk/> | 13 | 3 |
| Royal College of Midwifery <https://www.rcm.org.uk/search-results/?query=fgm&page=1> | 67 | 4 |
| Royal College of General Practitioners https://www.rcgp.org.uk/ | 6 | 1 |
| Royal College of Psychiatrists <https://www.rcpsych.ac.uk/> | 2 | 0 |
| British Medical Association <https://www.bma.org.uk/> | 4 | 2 |
| The Royal College of Paediatrics and Child Health <https://www.rcpch.ac.uk/> | 9 | 2 |
| FGM National Clinical Group <http://www.fgmnationalgroup.org/> | 1 | 1 |
| Care Quality Commission <https://www.cqc.org.uk/> | 95 | 0 |
| The National Institute for Health and Care Excellence <https://www.nice.org.uk/> | 5 | 4 |
| The National FGM Centre <http://nationalfgmcentre.org.uk/> | 3 | 3 |
| FGM Specialists Network <https://fgmnetwork.org.uk/services/fgm-support-services/> Resources +professions | 108 | 48 |
| **Total** | **2485** | **183** |
| Duplicates removed |  | 67 |
| Removed at screening |  | 28 |
| **Included** |  | **88** |
|  |  |  |
| **USA: Websites and search terms** FGM/ female genital mutilation/ female genital cutting |  |  |
| Centres for Disease Control <https://www.cdc.gov/> | 23 | 1 |
| Department of Health and Human Services <https://www.usa.gov/federal-agencies/u-s-department-of-health-and-human-services> | 980 | 1 |
| [Alabama](https://www.bbc.com/news/world/us_and_canada/states/al) Department of Public Health <https://www.alabamapublichealth.gov/index.html> | 3 | 0 |
| Alaska Department of Health & Social Services <https://dhss.alaska.gov/Pages/default.aspx> | 0 | 0 |
| [Arizona](https://www.bbc.com/news/world/us_and_canada/states/az) Department of Health Services <https://www.azdhs.gov/> | 10 | 0 |
| [Arkansas](https://www.bbc.com/news/world/us_and_canada/states/ar) Department of Health <https://www.healthy.arkansas.gov/> | 1 | 0 |
| [California](https://www.bbc.com/news/world/us_and_canada/states/ca) Department of Public Health <https://www.cdph.ca.gov/> | 0 | 0 |
| Department of Health Care Services <https://www.dhcs.ca.gov/> | 2 | 0 |
| [Colorado](https://www.bbc.com/news/world/us_and_canada/states/co) Department of Public Health and Environment <https://cdphe.colorado.gov/> | 25 | 0 |
| Colorado Department of Health Care Policy & Financing <https://hcpf.colorado.gov/> | 0 | 0 |
| [Connecticut](https://www.bbc.com/news/world/us_and_canada/states/ct) Department of Public Health <https://portal.ct.gov/DPH> | 502 | 0 |
| [Connecticut](https://www.bbc.com/news/world/us_and_canada/states/ct) Health and Human services <https://portal.ct.gov/Services/Health-and-Human-Services> | 502 | 0 |
| [Delaware](https://www.bbc.com/news/world/us_and_canada/states/de) division of public health <https://dhss.delaware.gov/dhss/dph/index.html> | 93 | 0 |
| Delaware Department of Health and Social Services <https://dhss.delaware.gov/dhss/> | 93 | 0 |
| [Florida](https://www.bbc.com/news/world/us_and_canada/states/fl) Department of Health <http://www.floridahealth.gov/> | 79 | 0 |
| Georgia Department of Public Health <https://dph.georgia.gov/> | 2035 | 1 |
| Georgia Department of Community Health <https://dch.georgia.gov/> | 505 | 0 |
| The Hawai'i Department of Health <https://health.hawaii.gov/> | 282 | 0 |
| Idaho Department of Health and Welfare <https://healthandwelfare.idaho.gov/> | 1 | 0 |
| [Illinois](https://www.bbc.com/news/world/us_and_canada/states/il) Department of Public Health <https://dph.illinois.gov/> | 2 | 0 |
| **Illinois Department** of Human Services <https://www.dhs.state.il.us/page.aspx> | 3 | 0 |
| [Indiana](https://www.bbc.com/news/world/us_and_canada/states/in) **Department** of Health <https://www.in.gov/health/> | 51 | 0 |
| Iowa Department of Public Health <https://idph.iowa.gov/> | 4 | 0 |
| Iowa Department of Human Services <https://dhs.iowa.gov/> | 0 | 0 |
| Kansas Department of Health and Environment <https://www.kdheks.gov/> | 0 | 0 |
| Kentucky Cabinet for Health and Family Services <https://chfs.ky.gov/agencies/dph/Pages/default.aspx> | 1 | 0 |
| Louisiana Department of Health <https://ldh.la.gov/> | 4 | 0 |
| Maine Department of Health and Human Services <https://www.maine.gov/dhhs/> | 0 | 0 |
| Maryland Department of Health <https://health.maryland.gov/Pages/Home.aspx> | 0 | 0 |
| [Massachusetts](https://www.bbc.com/news/world/us_and_canada/states/ma) **Department** of Health <https://www.mass.gov/orgs/department-of-public-health> | 19 | 0 |
| Michigan Department of Health and Human Services <https://www.michigan.gov/mdhhs/> | 4 | 0 |
| Minnesota Department of Health <https://www.health.state.mn.us/> | 5 | 1 |
| Mississippi State Department of Health <https://msdh.ms.gov/> | 0 | 0 |
| Missouri Department of Health and Senior Services <https://health.mo.gov> | 721 | 0 |
| Montana Department of Public Health and Human Services <https://dphhs.mt.gov/> | 0 | 0 |
| [Nebraska](https://www.bbc.com/news/world/us_and_canada/states/ne) Department of Health and Human Services [https://www.nebraska.gov/featured/health/](%20https://dhhs.ne.gov%20) | 4 | 0 |
| [Nevada](https://www.bbc.com/news/world/us_and_canada/states/nv) Department of Health and Human Services <https://dhhs.nv.gov/> | 5 | 0 |
| [New Hampshire](https://www.bbc.com/news/world/us_and_canada/states/nh) Department of Health and Human Services <https://www.dhhs.nh.gov/> | 0 | 0 |
| [New Jersey](https://www.bbc.com/news/world/us_and_canada/states/nj) Department of Health <https://www.nj.gov/health/> | 4 | 0 |
| [New Mexico](https://www.bbc.com/news/world/us_and_canada/states/nm) Department of Health <https://www.nmhealth.org/> | 6 | 0 |
| [New York](https://www.bbc.com/news/world/us_and_canada/states/ny) State Department of Health <https://www.health.ny.gov/> | 4 | 1 |
| [North Carolina](https://www.bbc.com/news/world/us_and_canada/states/nc) Department of Public Health and Human Services <https://www.ncdhhs.gov/> | 0 | 0 |
| [North Dakota](https://www.bbc.com/news/world/us_and_canada/states/nd) Department of Health <https://www.health.nd.gov/> | 0 | 0 |
| Ohio Department of Health <https://odh.ohio.gov/wps/portal/gov/odh/home> | 20 | 0 |
| Oklahoma State Department of Health <https://oklahoma.gov/health.html> | 0 | 0 |
| [Oregon](https://www.bbc.com/news/world/us_and_canada/states/or) Health Authority <https://www.oregon.gov/oha/Pages/index.aspx> | 76 | 0 |
| Pennsylvania Department of Health <https://www.health.pa.gov/Pages/default.aspx> | 2 | 0 |
| Rhode Island Department of Health <https://health.ri.gov/> | 3 | 0 |
| South Carolina Department of Health and Environmental Control <https://scdhec.gov/> | 0 | 0 |
| South Dakota Department of Health <https://doh.sd.gov/> | 0 | 0 |
| Tennessee Department of Health <https://www.tn.gov/health.html> | 23 | 0 |
| Texas Department of State Health Services <https://dshs.texas.gov/> | 0 | 0 |
| Utah Department of Health <https://health.utahcounty.gov/> | 0 | 0 |
| [Vermont](https://www.bbc.com/news/world/us_and_canada/states/vt) Department of Health <https://www.healthvermont.gov/> | 20 | 0 |
| [Virginia](https://www.bbc.com/news/world/us_and_canada/states/va) Department of Health <https://www.virginia.gov/agencies/virginia-department-of-health/> | 0 | 0 |
| [Washington](https://www.bbc.com/news/world/us_and_canada/states/wa) State Department of Health <https://www.doh.wa.gov/> | 0 | 0 |
| [Washington DC](https://www.bbc.com/news/world/us_and_canada/states/dc) Health <https://dchealth.dc.gov/> | 3 | 0 |
| [West Virginia](https://www.bbc.com/news/world/us_and_canada/states/wv) Department of Health and Human Resources <https://dhhr.wv.gov/Pages/default.aspx> | 0 | 0 |
| [Wisconsin](https://www.bbc.com/news/world/us_and_canada/states/wi) Department of Health Services <https://www.dhs.wisconsin.gov/> | 0 | 0 |
| [Wyoming](https://www.bbc.com/news/world/us_and_canada/states/wy) Department of Health <https://health.wyo.gov/> | 12 | 0 |
| American Nurses Association <https://www.nursingworld.org/> | 20 | 0 |
| American College of Nurse-Midwives <https://www.midwife.org/> | 2 | 1 |
| The American College of Obstetricians and Gynecologists <https://www.acog.org/> | 10 | 1 |
| The American College of Pediatricians <https://acpeds.org/> | 67 | 1 |
| The American Academy of Pediatrics <https://www.aap.org/> | 4 | 1 |
| American Academy of Family Physicians <https://www.aafp.org/home.html> | 34 | 1 |
| The American College of Psychiatrists <https://www.acpsych.org/> | 0 | 0 |
| American College of Physicians <https://www.acponline.org/> | 27 | 0 |
| American Medical Association <https://www.ama-assn.org/> | 2 | 1 |
| The American Psychological Association <https://www.apa.org/> | 0 | 0 |
| Planned Parenthood <https://www.plannedparenthood.org/> | 2 | 0 |
| **Total** | **6300** | **11** |
| Duplicates removed |  | 1 |
| Removed at screening |  | 1 |
| **Included** |  | **9** |
|  |  |  |
|  |  |  |
| **TOTAL in search** | **12655** |  |
| **Total duplicates** |  | **85** |
| **Total removed at screening** |  | **37** |
