# Supplementary material for: Woman and girl-centred care for those affected by female genital mutilation: a scoping review of provider tools and guidelines
Source: Reprod Health. 2022 Feb 22;19:50. doi: 10.1186/s12978-022-01356-3 (PMC8862274; doi:10.1186/s12978-022-01356-3)
Supplement: Supplementary file 2 — Additional file 2: Inclusion and exclusion criteria. [file 12978_2022_1356_MOESM2_ESM.docx]

**Additional file 2. Inclusion and exclusion criteria**

| **Inclusion criteria** | **Exclusion criteria** |
| --- | --- |
| Guidance statements and tools (algorithms, flip charts, etc.), technical guidelines, procedural documents and clinical practice guidelines | Training materials |
| < 20 years old (2001-2021) | >20 years old (before 2001) |
| High-income country settings | upper-middle-income, low or lower-middle-income country settings |
| Anglosphere countries | Non-English-speaking countries |
| English language | Languages other than English |
