# Supplementary material for: Woman and girl-centred care for those affected by female genital mutilation: a scoping review of provider tools and guidelines
Source: Reprod Health. 2022 Feb 22;19:50. doi: 10.1186/s12978-022-01356-3 (PMC8862274; doi:10.1186/s12978-022-01356-3)
Supplement: Supplementary file 3 — Additional file 3: Quality of included guidelines appraised with AGREE II. [file 12978_2022_1356_MOESM3_ESM.docx]

**Additional file 3. Quality of included guidelines appraised with AGREE II**

|  | **Domain Score %** | | | | | | (Average score) | Recommended for use |
| --- | --- | --- | --- | --- | --- | --- | --- | --- |
|  |  |  |  |  |  |  |  |  |
|  | Scope & purpose | Stakeholder involvement | Rigour of development | Clarity of presentation | Applicability | Editorial independence |  |  |
| **Australia** |  | | | | | | | |
| 1. (ACT Government 2017) Clinical guideline | 88.9 | 50.0 | 36.8 | 74.1 | 48.6 | 55.6 | 59.0 | Yes with edits |
| 1. (Adams and Adam 2001) Care of women with FGM | 83.3 | 42.6 | 22.9 | 87.0 | 59.7 | 16.7 | 52.0 | No |
| 1. (AMA 2017) Position Statement | 66.7 | 55.6 | 16.7 | 61.1 | 12.5 | 0.0 | 35.4 | No |
| 1. (Cancer Council Victoria, PapScreen Victoria et al. 2015) Statement on asking about FGM, supporting women & undertaking a pap smear | 88.9 | 46.3 | 22.9 | 75.9 | 34.7 | 50.0 | 53.1 | No |
| 1. (Chaves, Paxton et al. 2016) Recommendations for comprehensive post-arrival health clinical assessment for people from refugee-like backgrounds includes FGM | 100.0 | 70.4 | 26.4 | 85.2 | 43.1 | 55.6 | 63.4 | Yes with edits |
| 1. (Cohealth 2016) Example of clinician starting a conversation about FGM in an animated video | 88.9 | 48.1 | 26.4 | 81.5 | 41.7 | 50.0 | 56.1 | Yes with edits |
| 1. (Department of Health and Human Services 2004) Includes Practice Guidelines | 77.8 | 40.7 | 18.1 | 77.8 | 37.5 | 16.7 | 44.8 | No |
| 1. (Department of Health and Community Services 2006) Includes Practice Guidelines | 92.6 | 74.1 | 30.6 | 88.9 | 54.2 | 41.7 | 63.7 | Yes with edits |
| 1. (Family Planning Victoria 2012) Care plan | 100.0 | 66.7 | 0.0 | 66.7 | 12.5 | 0.0 | 41.0 | No |
| 1. (Government of Western Australia 2016) safeguarding & care guide | 40.7 | 13.0 | 19.4 | 70.4 | 27.8 | 33.3 | 34.1 | No |
| 1. (Homed 2014) Mandatory reporting tool to support health professionals | 0.0 | 0.0 | 8.3 | 66.7 | 8.3 | 50.0 | 22.2 | No |
| 1. (Jordan and Neophytou 2012) Service co-ordination guide that includes the flow chart outlining care | 100.0 | 74.1 | 12.5 | 88.9 | 75.0 | 33.3 | 64.0 | Yes with edits |
| 1. (King Edward Memorial Hospital 2015) Clinical practice guideline | 100.0 | 22.2 | 20.8 | 77.8 | 33.3 | 33.3 | 47.9 | No |
| 1. (NSW Health 2014) Practice Guidelines Maternity-Pregnancy and Birthing Care for Women | 100.0 | 72.2 | 50.0 | 83.3 | 41.7 | 33.3 | 63.4 | Yes with edits |
| 1. (NSW Health 2014) | 92.6 | 27.8 | 50.0 | 79.6 | 50.0 | 55.6 | 60 | Yes with edits |
| 1. (SA Maternal Neonatal & Gynacology Community of Practice 2018) Perinatal Practice Guideline | 83.3 | 55.6 | 25.0 | 66.7 | 33.3 | 50.0 | 52.3 | No |
| 1. (Queensland Clinical Guidelines 2018) Perineal care include deinfibulation for FGM | 83.3 | 55.6 | 27.1 | 66.7 | 41.7 | 50.0 | 54.1 | No |
| 1. (RACP 2012) Position Statement Physicians | 33.3 | 33.3 | 8.3 | 66.7 | 8.3 | 0.0 | 25.0 | No |
| 1. (RANZCOG 2017) Position Statement Obstetrics and Gynaecology | 72.2 | 83.3 | 35.4 | 77.8 | 41.7 | 58.3 | 61.5 | Yes with edits |
| 1. (The Royal Women's Hospital 2020) Clinical guideline | 66.7 | 50.0 | 20.8 | 66.7 | 50.0 | 50.0 | 50.7 | No |
| **Canada** |  |  |  |  |  |  |  |  |
| 1. (Canadian Paediatric Society 2018) Clinical guide | 77.8 | 50.0 | 24.3 | 63.0 | 36.1 | 50.0 | 50.2 | No |
| 1. (College of Physicians and Surgeons of Ontario 2011) Policy statement | 83.3 | 59.3 | 29.2 | 68.5 | 47.2 | 16.7 | 50.7 | No |
| 1. (Perron, Senikas et al. 2013) **clinical practice guideline developed by multiple professional committees** | 100.0 | 83.3 | 76.4 | 87.0 | 70.8 | 44.4 | 77.0 | Yes with edits |
| **Ireland** |  |  |  |  |  |  |  |  |
| 1. (IOG RCPI 2019) | 92.6 | 27.8 | 50.0 | 79.6 | 50.0 | 55.6 | 60 | Yes with edits |
| 1. (RCSI Health Service Executive AkiDwA 2013) Handbook for health professionals | 75.9 | 57.4 | 37.5 | 68.5 | 43.1 | 50.0 | 55.4 | Yes with edits |
| **New Zealand** | | | | | | | | |
| 1. (FGM Education Programme 2009) ANC, birth & PNC guidelines | 33.3 | 33.3 | 8.3 | 66.7 | 16.7 | 0.0 | 26.4 | No |
| 1. (FGM Education Programme 2009) De-infibulation guidelines | 33.3 | 22.2 | 8.3 | 33.3 | 8.3 | 0.0 | 17.6 | No |
| **United Kingdom** | | | | | | | | |
| 1. (Bracknell Forest Council 2017) *Care pathway flowchart for adults- pregnant and non-pregnant* | 68.5 | 22.2 | 16.0 | 72.2 | 29.2 | 50.0 | 43.0 | No |
| 1. (Bracknell Forest Council 2017) *Referral pathway flowchart for children* | 66.7 | 33.3 | 13.2 | 33.3 | 16.7 | 0.0 | 27.2 | No |
| 1. (Bristol Safeguarding Children Board 2011) *Guidance, referral risk assessment checklist tool & flowchart, key phases for interacting with a woman* | 88.9 | 35.2 | 43.8 | 83.3 | 44.4 | 50.0 | 57.6 | Yes with edits |
| 1. (Cambridgeshire & Peterborough SCPG 2017) *Interactive website with letter leaflet for professionals* | 72.2 | 27.8 | 18.8 | 81.5 | 30.6 | 50.0 | 46.8 | No |
| 1. (Carderdale Safeguarding 2017) *Care pathway flowcharts women, reporting & referral girls & women* | 100.0 | 72.2 | 36.8 | 66.7 | 48.6 | 50.0 | 62.4 | Yes with edits |
| 1. (Coventry City Council 2018) *Referral risk assessment checklist tool, introductory questions and guide to talking about FGM* | 88.9 | 57.4 | 23.6 | 81.5 | 47.2 | 50.0 | 58.1 | Yes with edits |
| 1. (Department of Health 2016) safe guidance Risk and Safeguarding: Guidance for professionals Risk assessment tables, suggested questions. | 96.3 | 77.8 | 59.7 | 85.2 | 62.5 | 5.6 | 64.5 | Yes with edits |
| 1. (Department of Health 2017) quick guide Quick guide Safeguarding and Risk Assessment: Risk assessment checklist, pointers for starting a conversation Serves as a guide for others | 33.3 | 22.2 | 0.0 | 66.7 | 16.7 | 0.0 | 23.1 | No |
| 1. (Department of Health 2017) Risk assess templates Practical tools | 55.6 | 22.2 | 27.1 | 74.1 | 51.4 | 16.7 | 41.2 | No |
| 1. (Department of Health 2017) Safeguarding pathway tables of checklists | 33.3 | 33.3 | 0.0 | 72.2 | 16.7 | 0.0 | 25.9 | No |
| 1. (Department of Health 2017) Multi-agency mandatory reporting duty Flowchart of reporting requirements & FAQs | 44.4 | 44.4 | 25.0 | 66.7 | 8.3 | 0.0 | 31.5 | No |
| 1. (Edinburgh Council, NHS Lothian et al. 2017) Inter-agency *Risk assessment & discussion topic guide tables, referral pathways for girls & women, dot points on asking questions* | 100.0 | 61.1 | 16.7 | 83.3 | 25.0 | 0.0 | 47.7 | No |
| 1. (Enfield Local Safeguarding Children Board 2014) | 66.7 | 38.9 | 0.0 | 33.3 | 8.3 | 0.0 | 24.5 | No |
| 1. (Hammersmith & Fulham 2019) Multi-agency *guidance talking about FGM & appropriate professional response* | 85.2 | 66.7 | 32.6 | 81.5 | 33.3 | 50.0 | 58.2 | Yes with edits |
| 1. (Hampshire SGAB 2016) Multi-agency *guidance talking about FGM & appropriate professional response* | 100.0 | 66.7 | 41.7 | 100.0 | 37.5 | 50.0 | 66.0 | Yes with edits |
| 1. (Harrow Council 2016) Multi-agency *report of situation in CCG & risk assess forms & pathways* | 61.1 | 27.8 | 20.8 | 88.9 | 58.3 | 50.0 | 51.2 | No |
| 1. (Hertfordshire Coucil, Hertfordshire Police Authority et al. 2017) *Multi-agency pathways, screening, referral risk assessment checklist tools & flowchart, questions to explore risk* | 66.7 | 27.8 | 22.9 | 83.3 | 25.0 | 50.0 | 45.9 | No |
| 1. (HM Government 2020) Multi-agency statutory guidance - safeguarding | 96.3 | 70.4 | 32.6 | 88.9 | 66.7 | 33.3 | 64.7 | Yes with edits |
| 1. (Home Office 2019) Multi-agenc*y* safeguarding & mandatory reporting duty for professionals resources | 88.9 | 33.3 | 29.2 | 77.8 | 33.3 | 50.0 | 52.1 | No |
| 1. (Home Office 2020) Multi-agenc*y m*andatory reporting procedures | 100.0 | 100.0 | 25.0 | 72.2 | 50.0 | 50.0 | 66.2 | Yes with edits |
| 1. (Kent and Medway Council 2018) *Multi-agency operational Guidelines and detailed risk assessment flow chart* | 100.0 | 50.0 | 37.5 | 83.3 | 54.2 | 50.0 | 62.5 | Yes with edits |
| 1. (Kingston and Richmond LSCB 2019) *Multi-agency risk assessment reporting & Referral form*s | 66.7 | 72.2 | 29.2 | 83.3 | 33.3 | 50.0 | 55.8 | Yes with edits |
| 1. (Lambeth Safeguarding Children Board 2017) *Multi-agency pathways, screening, referral risk assessment checklist tools & flowchart, questions suggested for talking with parents & girls* | 100.0 | 100.0 | 58.3 | 83.3 | 50.0 | 66.7 | 76.4 | Yes with edits |
| 1. (Leeds Safegurading partnership 2018) *Flowchart for managing concerns* | 72.2 | 27.8 | 6.9 | 66.7 | 16.7 | 50.0 | 37.7 | No |
| 1. (Liverpool safeguarding Children Board 2014) *Multi-agency pathway for referral for childre*n | 88.9 | 72.2 | 22.9 | 66.7 | 50.0 | 50.0 | 58.4 | Yes with edits |
| 1. (London Borough of Newham 2018) *Protocol with referral flow charts* | 94.4 | 83.3 | 41.7 | 100.0 | 66.7 | 58.3 | 74.1 | Yes with edits |
| 1. (London Safeguarding Children Board 2017) *Statement of how to report & risk indicators* | 55.6 | 0.0 | 6.3 | 68.5 | 33.3 | 50.0 | 35.6 | No |
| 1. (Milton Keynes Safeguarding Children Board, Milton Keynes Council et al. 2016) *Multi-agency pathways, screening, risk assessment checklist tools & referral flowchart* | 94.4 | 61.1 | 25.0 | 66.7 | 16.7 | 0.0 | 44.0 | No |
| 1. (NHS Birmingham 2017) Guideline for management and reporting in girls under 18yrs and women over 18yrs pregnant and non-pregnant. Guidance on starting a conversation, risk assessment tool, referral tables modified guide, taken from the DoH | 72.2 | 27.8 | 27.1 | 83.3 | 50.0 | 0.0 | 43.4 | No |
| 1. (NHS Croydon 2013) mandatory Reporting and safeguarding guidance, referral risk assessment checklist tool, 4 C’s to begin conversations about FGM | 100.0 | 33.3 | 8.3 | 33.3 | 16.7 | 50.0 | 40.3 | No |
| 1. (NHS James Paget University Hospital 2016) mandatory Reporting and safeguarding policy, includes checklist of values of staff, checklists and flow charts | 88.9 | 44.4 | 25.0 | 66.7 | 54.2 | 50.0 | 54.9 | No |
| 1. (NHS North East London Foundation Trust 2015) Multi-Agency Strategy, mandatory reporting flow chart | 72.2 | 66.7 | 27.1 | 66.7 | 41.7 | 33.3 | 51.3 | No |
| 1. (NHS Sandwell and West Birmingham 2017) Guideline for risk assessment, mandatory reporting, protection orders | 88.9 | 44.4 | 4.2 | 66.7 | 16.7 | 50.0 | 45.1 | No |
| 1. (NHS West Suffolk Hospital 2017) Safeguarding Adults at Risk of Abuse and Neglect | 61.1 | 77.8 | 27.1 | 72.2 | 41.7 | 66.7 | 57.8 | Yes with edits |
| 1. (Norfolk Safeguarding Children Partnership 2016) *Referral & assessment policy and procedures* | 50.0 | 5.6 | 14.6 | 66.7 | 0.0 | 50.0 | 31.1 | No |
| 1. (North East FGM Partnership Board 2017) *Multi-agency pathways, screening, risk assessment checklist tools* | 83.3 | 72.2 | 31.3 | 66.7 | 41.7 | 50.0 | 57.5 | Yes with edits |
| 1. (Northamptonshire Safeguarding Children Board 2016) *Risk assessment checklist tools* | 83.3 | 0.0 | 0.0 | 16.7 | 12.5 | 50.0 | 27.1 | No |
| 1. (Nottinghamshire NSCP 2019) *Reporting guidance* | 44.4 | 11.1 | 12.5 | 66.7 | 4.2 | 50.0 | 31.5 | No |
| 1. (Peterborough Safeguarding Children Board and LSCB 2017) *Referral flowchart for pregnant women & girls questions suggested for talking with parents & girls* | 88.9 | 44.4 | 4.2 | 66.7 | 16.7 | 50.0 | 45.1 | No |
| 1. (Public Health England 2016) Health visitors and school nurses mandatory reporting | 83.3 | 68.5 | 31.3 | 77.8 | 48.6 | 50.0 | 60 | Yes with edits |
| 1. (Public Health England 2016) Risk and safeguarding: guidance for nurses | 66.7 | 72.2 | 27.1 | 83.3 | 37.5 | 50.0 | 56.1 | Yes with edits |
| 1. (Islington Council 2015) Islington Risk Assessment tool | 100.0 | 77.8 | 29.2 | 77.8 | 16.7 | 50.0 | 58.6 | Yes with edits |
| 1. (Scottish Government 2017) Multi-agency pathways, screening, risk assessment checklist tools &referral flowcharts for women & girls based on the DoH Guide, notes importance of sensitive questioning points | 85.2 | 85.2 | 23.6 | 87.0 | 54.2 | 47.2 | 63.7 | Yes with edits |
| 1. (Southwark Children’s Safeguarding Board and Southwark Safeguarding Adults Board 2016) *Multi-agency pathways, screening, referral risk assessment checklist tools & flowchart, & questions* | 44.4 | 66.7 | 37.5 | 66.7 | 16.7 | 50.0 | 47.0 | No |
| 1. (Stoke-on-Trent and Staffordshire Safeguarding Children Board 2011) | 88.9 | 44.4 | 4.2 | 66.7 | 16.7 | 50.0 | 45.1 | No |
| 1. (Sunderland City Council 2016) | 88.9 | 44.4 | 25.0 | 66.7 | 54.2 | 50.0 | 54.9 | No |
| 1. (Sutton Council 2016) *Referral pathway flowchart for children & adults* | 16.7 | 11.1 | 0.0 | 33.3 | 1.4 | 0.0 | 10.4 | No |
| 1. (Surrey Safeguarding Children Partnership 2018) *Quick guide with interactive map* | 16.7 | 5.6 | 0.0 | 16.7 | 0.0 | 50.0 | 14.8 | No |
| 1. (Telford & Wrekin Council 2011) *Dot points on talking with girls and need for sensitivity. Section on awareness raising &* community education | 66.7 | 44.4 | 29.2 | 66.7 | 41.7 | 50.0 | 49.8 |  |
| 1. (Welsh Government 2019) Short guidance on communicating with victims mandatory reporting & safeguarding | 66.7 | 44.4 | 16.7 | 66.7 | 16.7 | 50.0 | 43.5 | No |
| 1. (Welsh Government 2011) Good practices for different sectors Multi-agency child protection protocol FGM | 66.7 | 44.4 | 22.9 | 66.7 | 41.7 | 33.3 | 45.9 | No |
| 1. (Wessex LMC 2018) Reporting & referral requirements for NHS staff | 16.7 | 16.7 | 0.0 | 33.3 | 8.3 | 50.0 | 20.8 | No |
| 1. (West Berkshire LSCB, Reading Local Safeguarding Children Board et al. 2016) *Multi-agency pathways for screening, referral, risk assessment checklist tools & flowchart* | 33.3 | 22.2 | 0.0 | 33.3 | 8.3 | 50.0 | 24.5 | No |
| 1. (Department of Health 2018) Recommendations for commissioners to make sure services meet the needs of women and girls with FGM and to safeguard those at risk | 83.3 | 100.0 | 0.0 | 83.3 | 70.8 | 83.3 | 70.1 | Yes with edits |
| 1. (FGM National Clinical Group 2013) Clinical standards for services includes referral & safeguarding, data collection & documentation | 83.3 | 77.8 | 47.9 | 63.0 | 45.8 | 50.0 | 51.3 | Yes with edits |
| 1. (NHS Ashford & St Peters Hospital 2016) *Management, Prevention and Detection of FGM in Women and Children, mandatory reporting and pregnancy pathways, flowchart*s | 88.9 | 50.0 | 16.7 | 66.7 | 33.3 | 58.3 | 52.3 | No |
| 1. (NHS Croydon 2011) Care pathway for pregnant women with FGM, suggested questions and mandatory Reporting and safeguarding | 77.8 | 50.0 | 27.1 | 66.7 | 33.3 | 50.0 | 50.8 | No |
| 1. (NHS Ipswich East Suffolk 2016) Guideline for management and reporting in girls under 18yrs and women over 18yrs | 77.8 | 72.2 | 35.4 | 66.7 | 33.3 | 0.0 | 47.6 | No |
| 1. (NHS Yeoville Hospital 2016) Obstetric Guideline and flow chart, paediatric safeguarding | 83.3 | 38.9 | 30.6 | 66.7 | 8.3 | 50.0 | 46.3 | No |
| 1. (Scottish Government 2016) Care pathway flowcharts women & girls risk assessment & referral | 66.7 | 44.4 | 29.2 | 66.7 | 33.3 | 50.0 | 48.4 | No |
| 1. (NHS Leichester 2015) *mandatory reporting flow chart and decision-making pathways for risk assessment and referral, suggestions for questions and medical record update* | 66.7 | 33.3 | 6.3 | 66.7 | 8.3 | 50.0 | 38.5 | No |
| 1. (NHS Leeds 2019) Standard Operating Procedure for recording and reporting | 77.8 | 50.0 | 35.4 | 66.7 | 33.3 | 0.0 | 43.9 | No |
| 1. (Barking Havering & Redbridge Hospitals NHS Trust 2012) | 88.9 | 50.0 | 16.7 | 66.7 | 33.3 | 58.3 | 52.3 | no |
| 1. (NHS Greater Glasgow and Clyde 2016) *Care Pathway for Revision (deinfibulation) of FGM* | 83.3 | 27.8 | 18.8 | 66.7 | 0.0 | 50.0 | 41.1 | No |
| 1. (NHS Greater Glasgow and Clyde 2020) Sexual health clinic guidelines | 66.7 | 66.7 | 6.3 | 33.3 | 0.0 | 50.0 | 37.2 | No |
| 1. (NHS Hammersmith Hospital 2007) Obstetric and midwifery guideline | 72.2 | 55.6 | 22.9 | 66.7 | 33.3 | 0.0 | 41.8 | No |
| 1. (NHS Heart of England Trust 2016) Obstetric and midwifery guideline and flowcharts | 100.0 | 66.7 | 27.1 | 83.3 | 25.0 | 0.0 | 50.3 | No |
| 1. (NHS Highland 2017) Flow charts for responding to a woman and girls *Obstetric and midwifery* | 72.2 | 22.2 | 33.3 | 66.7 | 20.8 | 0.0 | 35.9 | No |
| 1. (NHS Ispwich Hospital 2015)  *Obstetric and midwifery guideline with data form* | 100.0 | 77.8 | 47.9 | 66.7 | 45.8 | 50.0 | 64.7 | Yes with edits |
| 1. (NHS Mid Essex Hospital Services 2018) Guideline for the management Obstetrics and Gynaecology | 100.0 | 83.3 | 47.9 | 83.3 | 54.2 | 58.3 | 71.2 | No |
| 1. (NHS Royal Cornwall Hospitals Trust 2018) Obstetric Guideline and flow chart | 83.3 | 66.7 | 29.2 | 66.7 | 16.7 | 50.0 | 52.1 | No |
| 1. (NHS University Hopsitals Leicester 2018) Guideline for the management Obstetrics and Gynaecology | 83.3 | 50.0 | 33.3 | 66.7 | 33.3 | 50.0 | 52.8 | No |
| 1. (Public Health Wales 2018) Table outlining clinical pathway for assessment, care & referral & checklists for girls & women | 55.6 | 11.1 | 12.5 | 55.6 | 25.0 | 0.0 | 26.6 | No |
| 1. (National FGM Centre 2016) Guidance and Assessment Tool for Social Workers | 83.3 | 77.8 | 25.0 | 83.3 | 50.0 | 0.0 | 53.2 | No |
| 1. (National FGM Centre 2016) online tool FAQ for Social Workers | 33.3 | 44.4 | 16.7 | 66.7 | 25.0 | 0.0 | 31.0 | No |
| 1. (National FGM Centre 2016) Toolkit How to do direct work on FGM with children, young people, parents and carers for Social Workers | 83.3 | 51.9 | 36.8 | 70.4 | 50.0 | 50.0 | 57.0 | Yes with edits |
| 1. (National FGM Centre 2016) Medical check guidance | 77.8 | 55.6 | 0.0 | 33.3 | 12.5 | 50.0 | 38.2 | No |
| 1. (Gardner M 2016) Talking to patients guide for general practitioners | 5.6 | 0.0 | 0.0 | 55.6 | 0.0 | 0.0 | 10.2 | No |
| 1. (RCGPs 2016) A clinical approach for GPs | 55.6 | 22.2 | 16.7 | 66.7 | 0.0 | 50.0 | 35.2 | No |
| 1. (RCEM 2017) Safeguarding flowchart & reporting tool & clinical referral flowchart for emergency medical specialists | 83.3 | 27.8 | 18.8 | 33.3 | 1.4 | 50.0 | 35.8 | No |
| 1. (RCOG 2015) Clinical Green top guide Obstetrics & Gynaecology | 72.2 | 38.9 | 16.7 | 66.7 | 25.0 | 50.0 | 44.9 | No |
| 1. (RCM 2015) Position statement of midwifery | 55.6 | 22.2 | 12.5 | 33.3 | 0.0 | 50.0 | 28.9 | No |
| 1. (RCM, RCN et al. 2013) Intercollegiate Recommendations for identifying, recording, and reporting nursing & midwifery | 83.3 | 72.2 | 39.6 | 66.7 | 75.0 | 66.7 | 67.2 | Yes with edits |
| 1. (RCN 2019) Resource for nursing and midwifery practice | 77.8 | 22.2 | 50.7 | 83.3 | 54.2 | 50.0 | 56.3 | Yes with edits |
| 1. (RCN 2016) Multi-agency safeguarding & ANC guidelines in travel health services | 83.3 | 72.2 | 37.5 | 66.7 | 16.7 | 50.0 | 54.4 | No |
| 1. (General Pharmacy Council 2017) Mandatory Reporting | 44.4 | 22.2 | 0.0 | 77.8 | 0.0 | 0.0 | 24.1 | No |
| 1. (BMA 2011) Caring for patients and safeguarding children | 92.6 | 27.8 | 31.3 | 79.6 | 50.0 | 55.6 | 56.1 | Yes with edits |
| 1. (Coho, Parra et al. 2019) Working Therapeutically with Survivors counsellors, psychologists | 100.0 | 81.5 | 52.1 | 87.0 | 62.5 | 44.4 | 71.3 | Yes with edits |
| **United States** | | | | | | | | |
| 1. (AAFP 2020) Policy Family Physicians | 48.1 | 51.9 | 14.6 | 66.7 | 2.8 | 25.0 | 34.8 | No |
| 1. (ACNM 2017) Position statement nurse-midwives | 83.3 | 63.0 | 41.7 | 66.7 | 40.3 | 50.0 | 57.5 | Yes with edits |
| 1. (ACOG 2019) Short Policy statement Obstetrics and Gynaecology | 33.3 | 59.3 | 6.3 | 59.3 | 16.7 | 44.4 | 36.5 | No |
| 1. (AMA 2017) Short Policy statement | 66.7 | 16.7 | 18.1 | 63.0 | 9.7 | 44.4 | 36.4 | No |
| 1. (AAP 2010) Short Policy statement for paediatrics | 83.3 | 114.8 | 36.1 | 74.1 | 37.5 | 55.6 | 66.9 | Yes with edits |
| 1. (CDC 2020) SRH Screening during the Domestic Medical Examination for Newly Arrived Refugees | 88.9 | 70.4 | 36.8 | 81.5 | 41.7 | 55.6 | 62.5 | Yes with edits |
| 1. (Georgia Department of Public Health 2019) | 77.8 | 50.0 | 24.3 | 63.0 | 36.1 | 50.0 | 50.2 | No |
| 1. (NY State Health Department 2016) | 72.2 | 55.6 | 22.9 | 66.7 | 33.3 | 0.0 | 41.8 | No |
| 1. (Young, Nour et al. 2020) clinical report guidance for the clinician in paediatric care | 77.8 | 72.2 | 43.8 | 79.6 | 55.6 | 61.1 | 65.0 | Yes with edits |

AAFP. (2020). "Female genital mutilation." Retrieved 23 December, 2019, from <https://www.aafp.org/about/policies/all/genital-mutilation.html>.

AAP (2010). "Ritual Genital Cutting of Female Minors." Pediatrics **125**(5): 1088.

ACNM. (2017). "Female Genital Mutilation/Cutting." Retrieved 24 December, 2019, from <https://www.midwife.org/acnm/files/ACNMLibraryData/UPLOADFILENAME/000000000068/FemaleGenitalMutilationCuttingMay2017.pdf>.

ACOG (2019). College Statement of Policy Female Genital Mutilation. Washington, DC, The American College of Obstetricians and Gynecologists.

ACT Government (2017). Canberra Hospital and Health Services Clinical Guideline Female Genital Cutting (FGC) (applicable to adults and/or children). Canberrra, Canberra Hopsital and Health Srevices, ACT Government.

Adams, J. and M. Adam (2001). Mama & Nunu (Mother and Baby) Pregnancy Care for African Women: an Information Manual for Service Providers. Melbourne, Women’s Health West.

AMA (2017). Female Genital Mutilation, Australian Medical Association.

AMA (2017). Female Genital Mutilation Expansion of AMA Policy on Female Genital Mutilation H-525.980. Chicago, American Medical Association.

Barking Havering & Redbridge Hospitals NHS Trust (2012). Maternity Guideline for the Care of Women who have been affected by Female Genital Mutilation. Barking, NHS England.

BMA (2011). Female Genital Mutilation: Caring for patients and safeguarding children Guidance from the British Medical Association. London, British Medial Association

Bracknell Forest Council (2017). Bracknell Forest FGM Care Pathway for Adults. Bracknell Forest.

Bracknell Forest Council (2017). FGM referral to the multi-agency safeguarding hub/ Thames Valley Police Bracknell Forest, Bracknell Forest Council, Bracknell Forest Local Safeguarding Children Board, .

Bristol Safeguarding Children Board (2011). Female Genital Mutilation Multi-agency Guidance. Bristol, Bristol Safeguarding Children Board.

Cambridgeshire & Peterborough SCPG. (2017). "Female Genital Mutilation." from <https://www.safeguardingcambspeterborough.org.uk/children-board/professionals/fgm-2/>

Canadian Paediatric Society. (2018). "Female genital mutilation/cutting." Retrieved 20/05/2019, from <https://www.kidsnewtocanada.ca/screening/fgm>.

Cancer Council Victoria, PapScreen Victoria and Women’s Health West (2015). Female genital cutting (FGC) & cervical screening: A guide for practitioners. Victoria.

Carderdale Safeguarding (2017). Female Genital Mutilation: Calderdale Strategic Response. UK, Carderdale Safeguarding Children Board, Calderdale Safeguarding Adults Board, West Yorkshire Police, Calderdale Community Safety Partnership, NHS.

CDC (2020). Sexual and Reproductive Health Screening during the Domestic Medical Examination for Newly Arrived Refugees <https://www.cdc.gov/immigrantrefugeehealth/guidelines/domestic/sexually-transmitted-diseases/index.html#fgmc>. Washington DC, Centers for Disease Control and Prevention, U.S. Department of Health & Human Services.

Chaves, N. J., G. Paxton, B.-A. Biggs, A. Thambiran, M. Smith, J. Williams, J. Gardiner and J. S. Davis (2016). Recommendations for comprehensive post-arrival health assessment for people from refugee-like backgrounds. Surry Hills, Australasian Society for Infectious Diseases and Refugee Health Network of Australia.

Cohealth. (2016). "Female genital cutting: starting conversations about female circumcision." from <https://www.cohealth.org.au/health-promotion/fgc/>.

Coho, C., S. Parra, L. Hussein and C. Laffy (2019). Female Genital Trauma: Guidelines for Working Therapeutically with Survivors of Female Genital Mutilation <https://manorgardenscentre.org/resources/1924_Female_Genital_Trauma_Report_Web.pdf>. London, Dhalia Project, The Centre for Psychosexual Health, National Council of Pschotherapists.

College of Physicians and Surgeons of Ontario (2011). Policy statement #2-11: Female Genital Cutting (Mutilation). Toronto, College of Physicians and Surgeons of Ontario.

Coventry City Council (2018). FGM Safeguarding and Risk Assessment Tool. Coventry City, Coventry City Council.

Department of Health (2016). Female Genital Mutilation Risk and Safeguarding: Guidance for professionals <https://www.gov.uk/government/publications/safeguarding-women-and-girls-at-risk-of-fgm>. London, Social Care Local Government and Care Partnerships, Children Families and Communities, Marternity and Starting well, Department of Health.

Department of Health (2017). Female Genital Mutilation (FGM) - Mandatory reporting duty <https://www.gov.uk/government/publications/fgm-mandatory-reporting-in-healthcare>.

Department of Health (2017). FGM Professional Guidance Forms: Risk assessment templates <https://www.gov.uk/government/publications/safeguarding-women-and-girls-at-risk-of-fgm>. London.

Department of Health (2017). FGM Safeguarding and Risk Assessment: Quick guide for health professionals <https://www.gov.uk/government/publications/safeguarding-women-and-girls-at-risk-of-fgm>. London.

Department of Health (2017). FGM safeguarding pathway <https://www.gov.uk/government/publications/safeguarding-women-and-girls-at-risk-of-fgm>. London.

Department of Health (2018). Commissioning services to support women and girls with female genital mutilation. London, Government of the United Kingdom.

Department of Health and Community Services (2006). Female Genital Mutilation Resource Manual for Health Professionals. Darwin, Northern Territory Government.

Department of Health and Human Services (2004). Female genital mutilation: information for health professionals. Tasmania, Tasmanian Government.

Edinburgh Council, NHS Lothian, Police Scotland, East Lothian Council, Midlothian and West Lothian Council (2017). Edinburgh and the Lothians Inter-Agency Procedures for the Protection of Girls and Women at Risk of Female Genital Mutilation (FGM).

Enfield Local Safeguarding Children Board (2014). Protocol for Female Genital Mutilation Of Children and Young People. Endfield.

Family Planning Victoria (2012). Improving the health care of women and girls affected by female genital mutilation/cutting: care plan flow chart. Melbourne.

FGM Education Programme (2009). Female Genital Mutilation Clinical Care - Deinfibulation Guidelines. Auckland, New Zealand Ministry of Health.

FGM Education Programme (2009). Female genital mutilation clinical care: Antenatal, labour & birth and postnatal guidelines, New Zealand Ministry of Health.

FGM National Clinical Group (2013). Clinical standards for FGM services <http://www.fgmnationalgroup.org/literature.htm>. London, FGM National Clinical Group.

Gardner M, K. J. (2016). "Talking to your patients about female genital mutilation." InnovAiT: Education and inspiration for general practice. **10**(5): 304-306.

General Pharmacy Council (2017). Female genital mutilation: mandatory duty for pharmacy professionals to report. London, General Pharamcy Council.

Georgia Department of Public Health (2019). State of Georgia Refugee Health Guidelines Manual. Atlanta Georgia Department of Public Health.

Government of Western Australia (2016). Female genital cutting/ mutilation: a guide for health professionals. Subiaco, North Metropolitan Health Service, Women and Newborn Health Services, Australian Red Cross Society.

Hammersmith & Fulham, K. C., Westminster LSCB,. (2019). "Female Genital Mutilation." from <https://www.rbkc.gov.uk/lscp/information-professionals-and-volunteers/harmful-practices>.

Hampshire SGAB (2016). Tackling Female Genital Mutilation (FGM) In Hampshire: A Partnership Approach 2016 – 2019, Hampshire Safe Guarding Adults Board.

Harrow Council (2016). A Review of Female Genital Mutilation In Harrow. London, Harrow Council.

Hertfordshire Coucil, Hertfordshire Police Authority, NHS Herts Valleys Clinical Commissioning Group, National FGM Centre, Herforshire Safeguarding Children Board and NHS East and North Hertfordshire Clinical Commissioning Group (2017). Hertfordshire Multi-agency FGM Pathways. Herford, Hertfordshire Coucil.

HM Government (2020). Multi-agency statutory guidance on female genital mutilation <https://assets.publishing.service.gov.uk/government/uploads/system/uploads/attachment_data/file/912996/6-1914-HO-Multi_Agency_Statutory_Guidance_on_FGM__-_MASTER_V7_-_FINAL__July_2020.pdf> United Kingdom.

Home Office (2019). Guidance Female genital mutilation: resource pack. London, UK Gov.

Home Office (2020). Mandatory Reporting of Female Genital Mutilation – procedural information <https://assets.publishing.service.gov.uk/government/uploads/system/uploads/attachment_data/file/573782/FGM_Mandatory_Reporting_-_procedural_information_nov16_FINAL.pdf>.

Homed, I. (2014). Female genital mutilation/cutting: a mandatory reporting tool to support health professionals. Victoria, Women’s Health West.

IOG RCPI (2019). National Clinical Guideline Management of Female Genital Mutilation (FGM)

Dublin, Institute of Obstetricians and Gynaecologists, Royal College of Physicians of Ireland and the Clinical Strategy and Programmes Division, Health Service Executive.

Islington Council (2015). Islington FGM Risk Assessment Tool <https://www.islingtoncs.org/node/7872>. Islington.

Jordan, L. and K. Neophytou (2012). Improving the health care of women and girls affected by female genital mutilation/cutting: a service coordination guide. Melbourne, Family Planning Victoria.

Kent and Medway Council (2018). Kent and Medway Female Genital Mutilation Operational Guidelines, Kent Safeguarding Children Board, Medway Safeguarding Children Board, and the Kent and Medway Safeguarding Adults Board. .

King Edward Memorial Hospital (2015). Clinical practice guideline: Female genital mutilation, Government of Western Australia, North Metropolitan Health Service, King Edward Memorial Hospital.

Kingston and Richmond LSCB (2019). Kingston and Richmond LSCB Female Genital Mutilation Policy Updated 2019, Kingston and Richmond Local Safeguarding Children Board.

Lambeth Safeguarding Children Board (2017). Lambeth Safegurding Children Board FGM Multi-Agency Guidance Document. Lambeth.

Leeds Safegurading partnership (2018). Workflow for managing concerns regarding Female Genital Mutilation for under 18 year olds.

Liverpool safeguarding Children Board (2014). Safeguarding Children from Abuse through Female Genital Mutilation (FGM). L. F. G. M. a. M. C. W. s. H. A. Group. Liverpool.

London Borough of Newham (2018). Female Genital Mutilation Protocol.

London Safeguarding Children Board (2017). Safeguarding children at risk of abuse through female genital mutilation (FGM).

Milton Keynes Safeguarding Children Board, Milton Keynes Council, Thames Valley Police and Milton Keynes Clinical Commission Group (2016). MKSCB Female Genital Mutilation Screening Tool. Milton Keynes.

National FGM Centre (2016). FGM Direct Work Toolkit How to do direct work on FGM with children, young people, parents and carers <http://nationalfgmcentre.org.uk/wp-content/uploads/2019/02/Direct-Work-Toolkit-JAN-19.pdf>. London, Barnardo's and the Local Government Association.

National FGM Centre (2016). FGM Good Practice Guidance and Assessment Tool for Social Workers <http://nationalfgmcentre.org.uk/wp-content/uploads/2018/10/FGM-Good-Practice-Guidance-for-Social-Workers.pdf>. London, Barnardo's and the Local Government Association.

National FGM Centre (2016). Medical Examination for FGM Good Practice <http://nationalfgmcentre.org.uk/wp-content/uploads/2019/01/Medical-Examination-FGM-Good-Practice.pdf>. London, Barnardo's and the Local Government Association.

National FGM Centre (2016). Online FGM Assessment Tool <http://nationalfgmcentre.org.uk/fgm-assessment-tool/>. London, Barnardo's and the Local Government Association.

NHS Ashford & St Peters Hospital (2016). Management, Prevention and Detection of Female Genital Mutilation (FGM) in Women and Children, Women’s Health Governance Group.

NHS Birmingham (2017). Birmingham Health Female Genital Mutilation (FGM) Risk Assessment Tool. Birmingham, Birmingham South Central Clinical Commissioning Group, Birmingham CrossCity Clinical commissioning Group.

NHS Croydon (2011). Guideline for the Mangement of Female Genital Mutilation (FGM), NHS.

NHS Croydon (2013). Croydon FGM Risk Assessment Tool. Croydon, Croydon Clinical Commissioning Group, NHS.

NHS Greater Glasgow and Clyde (2016). NHSGGC Care Pathway for Revision of Female Genital Mutilation (FGM) <https://www.nhsggc.org.uk/media/256423/nhsggc_care_pathway_for_revision_of_fgm.pdf>.

NHS Greater Glasgow and Clyde (2020). Female Genital Mutilation Sandyford Guidelines <https://www.sandyford.scot/media/3492/female-genital-mutilation-ceg-march-2020.pdf>. Glasgow, Scotland, Sandyford Sexual Health Services.

NHS Hammersmith Hospital (2007). FGM Guidelines. Hammersmith, NHS Hammersmith.

NHS Heart of England Trust (2016). Female Genital Mutilation (FGM) in Obstetrics and Gynaecology (V4).

NHS Highland (2017). Responding to Female Genital Mutilation in Highland <https://www.hvawp.scot.nhs.uk/wp-content/uploads/2017/11/Responding-to-Female-Genital-Mutilation-in-Highland-Oct-16.pdf>.

NHS Ipswich East Suffolk (2016). FGM Guidance for Professionals. Ipswich, NHS Ipswich East Suffolk Clinical Comminssioning Group.

NHS Ispwich Hospital (2015). FGM Obsteric and Midwifery Guideline.

NHS James Paget University Hospital (2016). FGM Reporting and Safeguarding Policy, NHS James Paget University Hospital.

NHS Leeds (2019). Female Genital Mutilation ( FGM ) - Standard Operating Procedure for recording and reporting. Leeds, NHS.

NHS Leichester (2015). Female Genital Mutilation (FGM) Decision Making Guidance and Pathways. Leichester.

NHS Mid Essex Hospital Services (2018). Mangement of Female Genital Mutilation. Mid Essex, Mid Essex Hospital Services.

NHS North East London Foundation Trust (2015). Multi-Agency Strategy to Tackle Female Genital Mutilation 2015-2018. London.

NHS Royal Cornwall Hospitals Trust (2018). Female Genital Mutilation/Cutting (FGM/C) – Obstetric Clinical Guideline. Cornwall, NHS Royal Cornwall Hospitals Trust.

NHS Sandwell and West Birmingham (2017). Policy and Procedures to Address Female Genital Mutilation <https://sandwellandwestbhamccg.nhs.uk/publications/miscellaneous-1/1564-fgm-policy>. . Birmingham, Sandwell & West Birmingham Clinical Commissioning Group.

NHS University Hopsitals Leicester (2018). Management of Women who have undergone FGM.

NHS West Suffolk Hospital (2017). Safeguarding Adults at Risk of Abuse and Neglect.

NHS Yeoville Hospital (2016). FGM Policy, Trust Safeguarding Committee.

Norfolk Safeguarding Children Partnership. (2016). "5.14 Female Genital Mutilation." from <https://www.norfolklscb.org/about/policies-procedures/5-14-female-genital-mutilation/>.

North East FGM Partnership Board (2017). Female Genital Mutilation (FGM) Multi Agency Guidance.

Northamptonshire Safeguarding Children Board (2016). FGM - Screening Tool. Northamptonshire, Northamptonshire Safeguarding Children Board.

Nottinghamshire NSCP (2019). Female Genital Mutilation. Nottingham, Nottinghamshire Safeguarding Children Partnership (NSCP), Nottingham City Safeguarding Children Partnership (NCSCP).

NSW Health (2014). Female Genital Mutilation/Cutting Talking with Families An Educational Resource. Sydney, Ministry of Health, Government of NSW.

NSW Health (2014). Maternity-Pregnancy and Birthing Care for Women Affected by Female Genital Mutilation / Cutting. Sydney, Ministry of Health, Government of NSW.

NY State Health Department (2016). Female Genital Mutilation/Female Circumcision Reference Card for Health Care Providers. New York, New York State Health Department.

Perron, L., V. Senikas, M. Burnett and V. Davis (2013). "Female Genital Cutting." J Obstet Gynaecol Can **35**(11): e1-e18.

Peterborough Safeguarding Children Board and C. LSCB (2017). Practice Guidance for practitioners on Female Genital Mutilation (FGM). Peterborough.

Public Health England. (2016). "PHE FGM Guidelines - Female Genital Mutilation risk and safeguarding: guidance for Nurses." Retrieved 22 June 2019, from <https://www.guidelines.co.uk/womens-health/phe-fgm-guideline/455186.article>.

Public Health England (2016). Understanding Female Genital Mutilation (FGM) Helping health visitors and school nurses tackle female genital mutilation. London, Public Health England.

Public Health Wales (2018). All Wales Clinical Pathway – Female Genital Mutilation. Cardiff, Public Health Wales.

Queensland Clinical Guidelines (2018). Perineal Care. Brisbane, Queensland Health.

RACP (2012). Female Genital Mutilation/Cutting. Sydney, Royal Australasian College of Physicians.

RANZCOG (2017). Female Genital Mutilation. Melbourne, Royal Australian & New Zealand College of Obstetricians and Gynaecologists.

RCEM (2017). A universal FGM flowchart and reporting tool. London, The Royal College of Emergency Medicine.

RCGPs (2016). Female Genital Mutilation: A clinical approach for GPs. London, Royal College of General Practitioners

RCM (2015). Position Statement Female Genital Mutilation. London, Royal College of Midwives.

RCM, RCN, RCOG, Equality Now and UNITE (2013). Tackling FGM in the UK: Intercollegiate Recommendations for identifying, recording, and reporting. London, Royal College of Midwives.

RCN (2016). Female Genital Mutilation RCN guidance for travel health services. London, Royal College of Nursing.

RCN (2019). Female Genital Mutilation An RCN resource for nursing and midwifery practice. London, Royal College of Nursing. **4th Edition**.

RCOG (2015). Female Genital Mutilation and its Management Green-top Guideline No. 53. London, Royal College of Obstetricians and Gynaecologists.

RCSI Health Service Executive AkiDwA (2013). Female Genital Mutilation: Information for Health-Care Professionals Working in Ireland <https://www.lenus.ie/handle/10147/306763> Dublin, AkiDwA.

SA Maternal Neonatal & Gynacology Community of Practice (2018). South Australian Perinatal Practice Guideline: Female Genital Mutilation. Adelaide, Department of Health and Wellbeing, Government of South Australia.

Scottish Government (2016). Guidance for service specification and standards for healthcare to prevent female genital mutilation (FGM) and respond to the needs of the survivors. Edinburgh, Chief Medical Officer Directorate, Directorate for Chief Nursing Officer, Patients, Public and Health ProfessionalsThe Scottish Government.

Scottish Government (2017). Responding to Female Genital Mutilation in Scotland: multi-agency guidance. Edinburgh.

Southwark Children’s Safeguarding Board and Southwark Safeguarding Adults Board (2016). Southwark Female Genital Mutilation Guidance Document for safeguarding children and vulnerable adults. Southwark.

Stoke-on-Trent and Staffordshire Safeguarding Children Board (2011). Guidance For Children Who May Be Particularly vulnerable Female Genital Mutilation. Stoke-on-Trent.

Sunderland City Council (2016). Female Genital Mutilation Multi-Agency Procedure. Sunderland, Sunderland Safeguarding Adults Board and Sunderland Safeguarding Children Board.

Surrey Safeguarding Children Partnership (2018). Surrey Safeguarding Children Partnership Procedures Manual 5.14 Female Genital Mutilation. Surrey, Surrey Safeguarding Children Partnership,.

Sutton Council (2016). Sutton FGM Pathway.

Telford & Wrekin Council (2011). FGM Practice Guidance & Resource Pack. Telford, Telford & Wrekin Council.

The Royal Women's Hospital (2020). Female genital mutilation/cutting - Guideline for Care. Melbourne, The Royal Women's Hospital.

Welsh Government (2011). All Wales Protocol FGM. Cardiff, All Wales Child Protection Procedures Review Group.

Welsh Government. (2019). "FGM Guidance for Professionals." Retrieved 28 December, 2019, from <https://gov.wales/female-genital-mutilation-guidance-professionals>.

Wessex LMC (2018). FGM Requirements for NHS Staff. Wessex, Wessex Local Medical Committees.

West Berkshire LSCB, Reading Local Safeguarding Children Board and Wokingham Safeguarding Children Board (2016). FGM Assessment Tool. West Berkshire.

Young, J., N. M. Nour, R. C. Macauley, S. K. Narang and C. Johnson-Agbakwu (2020). "Diagnosis, Management, and Treatment of Female Genital Mutilation or Cutting in Girls." Pediatrics **146**(2): e20201012.
